# Active-learning-guided optimization of cell-free systems for genome-wide transcriptomic profiling reveals progressive layers of regulation

**DOI:** 10.1038/s41467-026-74559-y

**Published:** 2026-06-27

**Authors:** Léa Wagner, An Hoang, Olivier Rue, Olivier Delumeau, Valentin Loux, Gabin Derache, Jean-Loup Faulon, Matthieu Jules, Olivier Borkowski

**Affiliations:** 1 Université Paris-Saclay, INRAE, AgroParisTech, Micalis Institute, Jouy-en-Josas, France; 2 Université Paris-Saclay, INRAE, MaIAGE, Jouy-en-Josas, France; 3 Université Paris-Saclay, INRAE, BioinfOmics, MIGALE bioinformatics facility, Jouy-en-Josas, France

**Keywords:** Synthetic biology, Machine learning, Gene expression profiling, Gene regulatory networks

## Abstract

Understanding genome regulation is limited by the complexity of molecular interactions in living cells. Cell-free systems provide a simplified platform for studying gene expression, but low mRNA levels have prevented RNA-seq. To address this, we develop an active learning workflow combining Bayesian optimization with automated high-throughput experimentation to systematically explore over 1.6 million buffer compositions, experimentally testing 653. We identify a “mRNA-optimized” buffer (20-fold increase in mRNA yield) and a “trade-off” buffer (13-fold increase while maintaining protein production). Using direct RNA-seq, we profile the T7 phage transcriptome in cell-free systems and compare it with a purified T7-RNAP transcription system and phage-infected bacteria. This comparative analysis reveals distinct regulatory layers: the T7-RNAP system captures promoter-strength hierarchies but lacks RNA degradation, whereas cell-free systems provide an accurate estimation of in vivo expression and reveal mRNA maturation sites. This work establishes cell-free transcriptomics as a controlled approach to study genome regulation.

## Abstract (web summary)

Cell-free is a powerful platform to unravel biological complexity. Here, the authors used active learning to optimize buffer composition, boosting mRNA 20-fold, enabling cell-free transcriptomics and uncovering progressive regulatory layers in T7 phage.

## Introduction

Lysate-based cell-free systems (CFS) are valuable platform for studying fundamental biological processes and molecular mechanisms^1–3^. Transcription and translation are restored in bacterial lysates by supplementing it with a pH-buffered mixture (called “buffer” hereafter) containing ATP regeneration substrates, crowding agents, salts, and essential precursors such as nucleotides and amino acids^4^. As such, CFS are simpler than living cells in terms of molecular interactions as they eliminate complex and interfering biological processes such as cell division and membrane-related functions. For example, a previous experiment showed that production and metabolic burdens can be untangled using cell-free compared with in vivo measurements^5^. CFS provides an open platform ideally suited for protein production, high-throughput prototyping of genetic circuits, biomanufacturing and gene expression analysis and modeling^6–8^.

While cell-free systems simplify the biochemical environment and enable direct manipulation of the transcriptional machinery, transcriptomic analyses have traditionally been performed in living cells to capture global gene regulation. RNA sequencing (RNA-seq) is a key method for studying gene expression in living cells^9^. For organisms like model bacteria, that are cultivable under laboratory conditions, transcriptomic analyses are relatively straightforward^10^. However, even with these organisms, complex regulatory cascades, growth-dependent effects, and intertwined regulatory processes often make it challenging to unambiguously identify the direct targets of specific regulators^11^. Furthermore, transcriptomic studies are particularly challenging for unculturable organisms. Metatranscriptomic approaches have been developed to address such limitations, but they remain limited by community-level signal convolution, higher limits of detection, and difficulties in resolving low-abundance, regulator-specific transcriptional responses^12^.

A few studies have used CFS to express entire genomes^13–15^. For example, Shin et al. used an *E. coli* lysate-based cell-free system to produce viable T7 phage virions from its genomic DNA, although transcriptomic analysis was not performed^13^. Taking a step further, Fujiwara et al. used CFS to express the genome of *Thermus thermophilus* and quantify the expression of a subset of genes using qPCR^16^. Despite these advances, one significant limitation of CFS is the relatively low yield of mRNA, which has prevented its use for high-throughput transcriptomic studies^16^.

To achieve mRNA abundances suitable for RNA-seq, the RNA-polymerase activity and mRNA stability in CFS can be enhanced by optimizing the buffer composition. We propose to tackle this optimization issue using active learning, an artificial intelligence method that makes use of machine learning algorithms to determine the next set of experiments to be carried out while studying a given problem^17^. Active learning has already been successfully applied to CFS data in order to optimize buffer composition and substantially increase protein yield^18–25^. As a proof of concept for cell-free transcriptomics, we focused on T7 RNA polymerase (T7 RNAP)-driven transcription to express the genome of bacteriophage T7 in lysate-based CFS. T7 RNAP is a highly processive and promoter-specific polymerase that provides a robust and well-controlled transcriptional system. The T7 genome (~40 kb) is compact, simply organized and well annotated, making it an ideal benchmark to evaluate genome-wide mRNA production in CFS.

In this work, we developed an active learning-guided optimization workflow combining Bayesian optimization with automated high-throughput experimentation, using an acoustic liquid handling system. In total, we measured the mRNA yield and protein yield across 653 unique buffer compositions, progressively refining our model and achieving a 20-fold increase in mRNA yield compared to the reference buffer. Using optimized CFS, we successfully expressed the genome of the 40 kb T7 phage genome in CFS and performed direct RNA sequencing (DRS) using a MinION sequencer from Oxford Nanopore Technologies. We demonstrated the relevance of cell-free transcriptomics to unravel the multiple layers of genome regulation by comparing the transcriptomic profiles obtained in CFS, in a minimalist in vitro transcription system and in vivo. Taken together, we present a proof of concept for cell-free transcriptomics, demonstrating that cell-free systems are powerful tools to investigate genome regulation in a controlled biochemical environment.

## Results

### High-throughput experimental framework to explore CFS composition

**Fig. 1:**
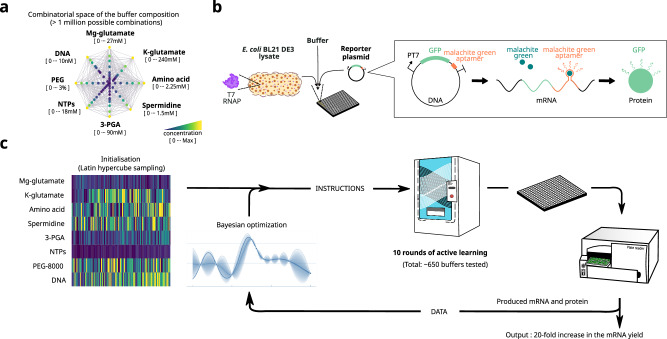
Active learning-based optimization of the cell-free buffer composition. **a** Combinatorial space of buffer composition in gray. The compounds and their maximum concentration range are indicated with the minimum concentration in blue and the maximum in yellow. **b** Schematic of the cell-free system setup: *E. coli* BL21 DE3 lysate is complemented with a buffer and a reporter plasmid. The reporter plasmid encodes *degfp* and malachite green aptamer under the control of the T7 promoter for measuring mRNA and protein abundances via fluorescence. The lysate from *E. coli* BL21 DE3 contains the T7 RNA polymerase produced during the culture growth. All measurements were performed in 384-well plates. **c** Active learning pipeline for buffer optimization. The process was initiated with 100 buffer combinations (each column matches with one buffer composition; for the 8 compounds, their concentration is indicated with the minimum in blue and the maximum in yellow), followed by 10 rounds of Bayesian optimization, resulting in a total of 653 unique buffer compositions tested.

The CFS “reference buffer” composition is based on the protocol developed by the Noireaux laboratory, omitting the non-essential components identified by Borkowski et al.^4,20^. It contains 4 mM Mg-glutamate, 120 mM K-glutamate, 1.5 mM of a mixture of the 20 amino acids, 1 mM spermidine, 30 mM 3-phosphoglyceric acid (3-PGA), 1.5 mM of a mixture of the four NTPs, 2% PEG-8000, and 50 mM HEPES. To optimize the CFS buffer for increased T7 RNAP-driven mRNA abundance, we explored a large combinatorial space. The combinatorial space is defined using six concentrations per component, anchoring the fifth at the reference level to enable exploration of four lower and one higher concentration (Fig. 1a). To monitor transcription and translation in CFS, we used a reporter plasmid encoding *degfp* and the malachite green aptamer under the control of a T7 promoter, enabling fluorescence-based measurements in a plate reader (Fig. 1b). Each cell-free reaction had a total volume of 10.5 µL and was composed of *E. coli* BL21 DE3 lysate containing T7 RNAP, the reporter plasmid, and a variable buffer composition (Fig. 1b). In order to compare measurements between plates, we maximized a normalized fluorescence metric called yield, defined as the fluorescence produced by a given buffer composition relative to that obtained with the reference composition. We implemented a high-throughput experimental workflow using an acoustic liquid handler (Echo 650, Beckman Colter, USA) for pipetting and a plate reader (Synergy HTX, BioTek, USA) for fluorescence measurements in 384-well plates (Fig. 1c). The limits of the combinatorial space were constrained by technical requirements of the Echo machine. Indeed, each ingredient’s stock concentration must be (i) high enough that the total volume of all ingredients remains below 10.5 µL, (ii) low enough to avoid exceeding solubility limits or causing excessive viscosity, and (iii) compatible with acoustic dispensing, such that each individual transfer falls within the 20-1500 nL range of the Echo liquid handler. Stock concentrations were selected to satisfy these criteria, and a drop test was performed to confirm that all ingredients could be reliably dispensed by the Echo 650 (Beckman Colter, USA). During optimization, the HEPES concentration was kept constant to avoid pH variations that could affect fluorescence measurements^26^. The lysate fraction was also kept constant. This initial combinatorial space comprises 1,679,616 possible buffer compositions.

### Evaluation of active learning models for optimizing composition

To efficiently explore a large combinatorial space of buffer compositions, we coupled automated experimental cycles with a Bayesian optimization framework, a powerful strategy for finding the extrema of a black-box objective function that is expensive to evaluate^27^. In this approach, a surrogate model is iteratively updated using experimental observations, and an acquisition function uses both predicted performance and uncertainty to guide the selection of new conditions to test. We first compared the predictive performance of several surrogate models, including a Multilayer Perceptron (MLP), Random Forest (RF), XGBoost, and Gaussian Process (GP) regression. Among these, the GP consistently showed the most robust performance on limited datasets and provided reliable uncertainty estimates, leading us to select it as the surrogate model for subsequent active learning cycles (see “Methods”, Supplementary Fig. 1a). We next evaluated different acquisition strategies, including Upper Confidence Bound (UCB), expected improvement (EI), and probability of improvement (PI) (Supplementary Fig. 1b). During the initial iterations (plates 0–2), UCB was used with a manually adjusted exploration-exploitation balance to rapidly identify high-performing regions of the parameter space. To directly assess whether additional optima could be revealed through exploration, plate 3 was designed to compare two strategies in parallel: half of the tested buffer compositions were selected using a purely exploitative strategy (EI), while the other half were selected using a purely exploratory UCB strategy. Since exploratory sampling did not yield improvements beyond model predictions (Supplementary Fig. 1c), all subsequent iterations (plate 4) relied mainly on exploitative sampling using EI.

### Active-learning-driven optimization to maximize mRNA level

**Fig. 2:**
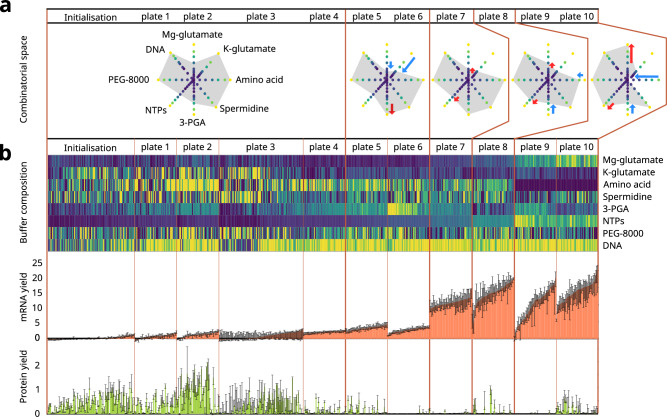
Iterative optimization of buffer composition increases mRNA yield by 20-fold (95% confidence interval of [16.45, 21.33]) after ten cycles. **a** Visualization of the combinatorial space explored on each plate, showing the distribution of buffer components across conditions. In each combinatorial space, red and blue arrows indicate increases and decreases, respectively, in the maximum concentration of a compound compared to the previous space. **b** Buffer optimization across 10 rounds of active learning. The heatmap gives the composition of the tested buffers, with the minimum (in blue) and the maximum (in yellow) tested concentrations. Coral and green bar plots show, respectively, the median mRNA and protein yields for each buffer composition. Error bars represent the 95% confidence interval across replicates (*n* = 3 for plates 0–3; *n* = 6 for plates 4–10). Source data are provided as a Source Data file.

Each iteration contained new cell-free buffer compositions tested in triplicate across plates 0–3. However, due to substantial variability in fluorescence measurements among replicates, we increased the number of replicates to six for plates 4–10 to improve measurement robustness. When yield plateaus were observed and could be attributed to component concentrations reaching the boundaries of the explored combinatorial space, the search space was expanded accordingly (gray area in Fig. 2a). For example, by the end of plate 6, mRNA yield did not exceed a fourfold improvement, and the top-performing 50 buffer compositions were strongly enriched for the highest NTPs concentrations within the defined space. We decided to increase the maximum concentration of NTPs in the combinatorial space. Moreover, based on the literature, we also increased the maximum concentration of Mg-glutamate^28^. These changes resulted in a significant increase in mRNA yield in plate 7 (Fig. 2a, b). Further expansion of the NTPs concentrations in plates 8–10 resulted in only marginal additional gains in mRNA abundance (Fig. 2a, b). In parallel, protein yield declined sharply after three iterations, with only a small subset of buffer compositions sustaining detectable protein production (Fig. 2b). As no substantial further improvement in mRNA yield was achieved, the active learning campaign was terminated after ten iterations. In total, 653 buffer compositions were tested, leading to a ~ 20-fold (95% confidence interval of [16.45, 21.33]) increase in mRNA yield in a composition termed the “mRNA-optimized buffer” (Fig. 2b). A 7.5-fold increase in mRNA amount was observed using mRNA purification in the mRNA-optimized buffer reaction, and a strong correlation between yield obtained by the malachite green and mRNA purification across multiple buffer compositions (Supplementary Fig. 2). As both methods carry inherent biases, the mRNA fold-improvement likely falls between these two values. Furthermore, mRNA purification strengthened our confidence in the rank ordering of buffer compositions relative to mRNA yield (Supplementary Fig. 2, Supplementary Fig. 3).

### The impact of buffer composition on mRNA yield

Testing 653 buffer combinations showed correlations between mRNA yield and the concentration of components, some of which confirmed by the scientific literature while others revealing novel observations (Supplementary Fig. 3). Our optimization consistently favored high concentrations of Mg-glutamate and NTPs, reflecting their synergistic roles as T7 RNA polymerase cofactor and substrate, respectively (Supplementary Fig. 4). This is consistent with the two-metal-ion model of transcription, in which two magnesium ions coordinate the nucleophilic attack of the 3’-OH on the α-phosphate of the incoming NTP, facilitating phosphodiester bond formation. As Mg^2+^ is chelated by nucleotides, the concentration of the two components must increase accordingly to avoid reducing T7 RNA polymerase efficiency^28,29^. A new finding is the optimal intermediate concentration of 3-PGA to maximize mRNA yield, the mechanism behind this observation will be investigated in future studies (Supplementary Fig. 4). In contrast, three compounds in the buffer, amino acids, spermidine, and PEG-8000, remained unconstrained by the active learning model suggesting that they have little influence on mRNA yield within the tested ranges (Supplementary Fig. 3). For amino acids, this result was expected as they are not required for transcription. Although spermidine has been reported as a critical component for in vitro transcription^30^, our data showed no benefit, which may be explained by the presence of endogenous polyamines in the lysate that compensate for its absence. Additionally, since PEG-8000 is designed to enhance crowding in the cell-free reaction, the increased concentrations of other components may have mitigated its effects. Overall, our active-learning-guided optimization simultaneously validates established transcription conditions and shows unintuitive optimal buffer compositions.

### Buffer optimization reveals mRNA and protein anticorrelation

**Fig. 3:**
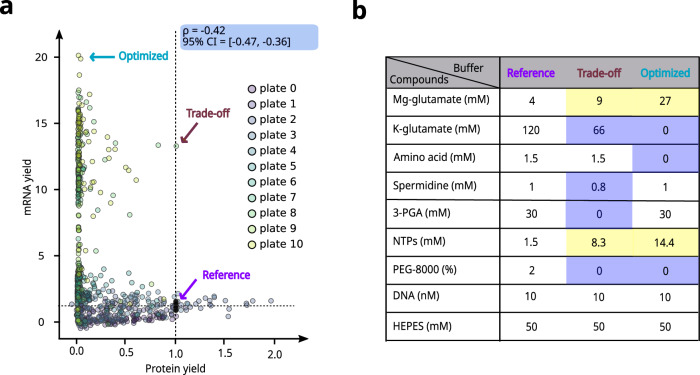
Optimal and trade-off buffer compositions and their corresponding mRNA and protein yields. **a** Scatter plot of protein yield versus mRNA yield for each buffer composition across plates. Each point represents one buffer condition. Highlighted are the reference buffer (purple), trade-off buffer (brown), and mRNA-optimized buffer (blue). Dashed lines indicate the reference yield (normalized to 1) for both mRNA and protein. The Spearman rank correlation coefficient (ρ) and its 95% confidence interval (CI), estimated by bootstrap resampling (*n* = 10,000), are shown. The reported ρ quantifies the monotonic relationship between mRNA and protein yields across all buffer compositions; the 95% CI indicates the uncertainty associated with this estimate. **b** Composition of the reference, trade-off and mRNA-optimized buffers. Compounds highlighted in yellow and in blue have increased and decreased concentrations compared to the reference, respectively. HEPES concentration was fixed during optimization. Source data are provided as a Source Data file.

Most compositions which favored mRNA yields resulted in reduced protein yields, consistent with previous observations^31^ (Fig. 3a, Supplementary Fig. 3). However, a “trade-off” buffer composition demonstrated that high mRNA abundance does not necessarily come with low protein production (Fig. 3a). Moreover, as the optimization algorithm concentrated on regions predicted to enhance mRNA yield large regions of the combinatorial space still remains underexplored. This partial coverage was also influenced by the intentional reduction of amino acid concentrations in plates 9 and 10, based on their minimal impact on mRNA output in earlier rounds. Several regions remain underexplored, including combinations of low DNA with elevated NTPs or Mg-glutamate, elevated amino acids with high NTPs, high K-glutamate with either elevated NTPs or Mg-glutamate and elevated NTPs with low Mg-glutamate (Supplementary Fig. 5). Notably, a high K-glutamate concentration has an important role in ribosome structure and function^32^. Thus, although high protein yield can reduce mRNA yield, we cannot conclusively determine whether competition between translation and transcription limits protein production. If such competition exists, some compounds might appear beneficial not because they directly enhance transcription, but because they reduce translation, an energetically costly process^33^. Eventually, for genome-wide transcriptomic analysis, two buffer compositions were selected: the “mRNA-optimized” buffer and the “trade-off” buffer with a 13-fold increase in mRNA yield but comparable protein abundance with the reference composition. The mRNA-optimized buffer contains higher levels of Mg-glutamate and NTPs, but lower levels of K-glutamate, amino acids, and PEG-8000 compared with the reference buffer (Fig. 3b). A similar trend is observed for the trade-off buffer, which shows a 13-fold improvement over the reference buffer, except that it maintains a comparable amino acid concentration and exhibits no 3-PGA and lower spermidine levels (Fig. 3b).

### Experimental workflow for genome-wide cell-free transcriptomics

**Fig. 4:**
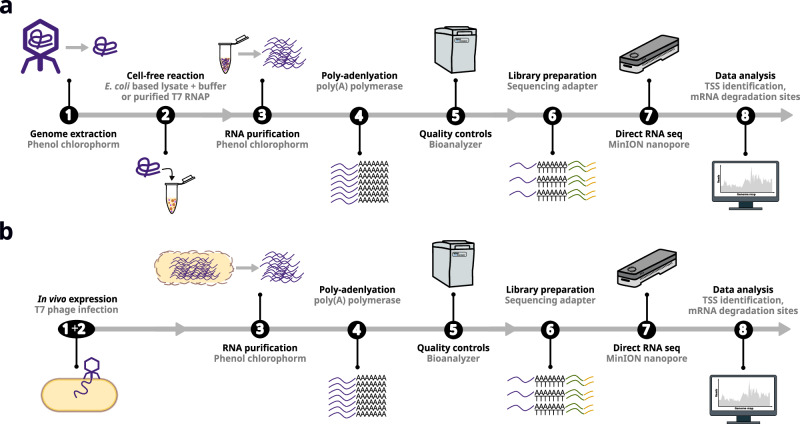
Schematic of the experimental transcriptomics workflows for T7 phage transcriptome analysis. **a** Cell-free experimental transcriptomics workflow. Direct RNA nanopore sequencing was performed using an eight-step workflow: (1) T7 genomic DNA extraction; (2) supplementation of the optimized cell-free reaction with the T7 genome and determination of the optimal RNA harvest time using a parallel reporter reaction; (3) total RNA isolation; (4) polyadenylation; (5) RNA integrity assessment; (6) library preparation; (7) real-time direct RNA sequencing on a MinION flow cell; and (8) data analysis (see “Methods”, Supplementary Fig. 6). **b** In vivo experimental transcriptomics workflow. T7 phage-infected *E. coli* cells served as the expression system (step 1 + 2), followed by RNA extraction and processing as described above.

**Fig. 5:**
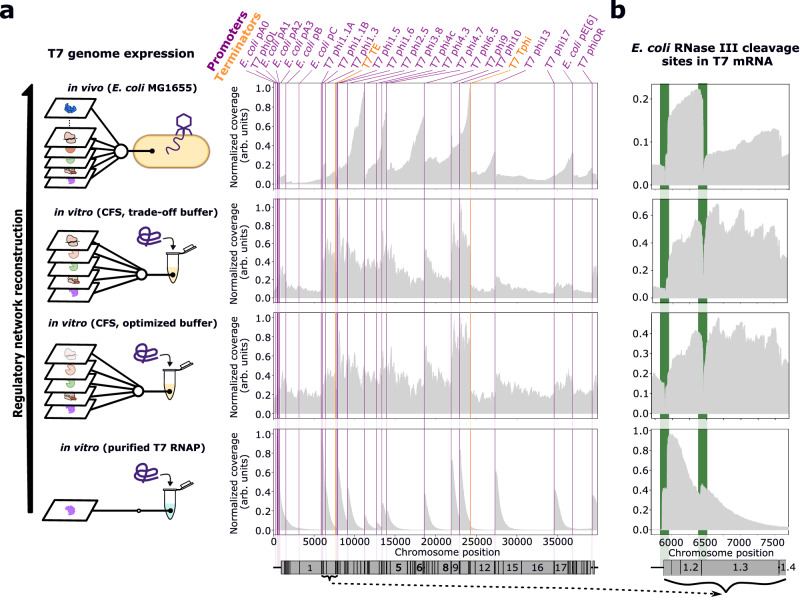
Comparative transcriptomics of T7 genome using a cell-free system. **a** Genome-wide expression profile of the T7 genome in various experimental conditions. On the left, a schematic of the T7 genome expression in vivo and in cell-free using the trade-off and mRNA-optimized buffers, as well as with purified T7 RNAP. In cell-free reactions using the mRNA-optimized buffer, translation is inactive because essential components, including amino acids and K-glutamate, are absent; the *E. coli* ribosome is shown in a transparent form to indicate lack of activity. On the right, sequencing depth (i.e., the number of times each base in the genome has been sequenced), normalized by the maximum value within each sample, is indicated by gray shaded areas. Promoters and terminators are shown in purple and orange, respectively. The position of the genes on the chromosome is represented in gray under the plots. Replicates of these experiments are presented in Supplementary Fig. 7. **b** Coverage plot highlighting genes 1.2 and 1.3, illustrating the activity of RNase III at known cleavage sites in T7 mRNA (in green). Source data are provided as a Source Data file.

**Fig. 6:**
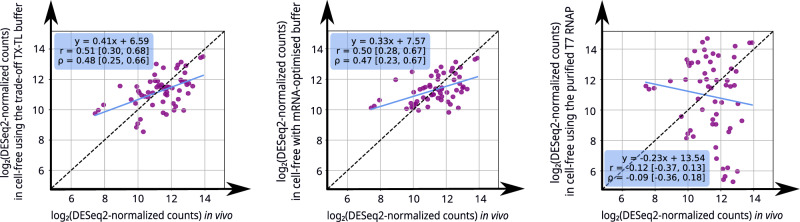
Correlation of log₂ DESeq2-normalized gene counts between in vivo expression and cell-free conditions. Scatter plots show pairwise comparisons of log₂-transformed DESeq2-normalized gene counts between in vivo infection and the trade-off buffer (left), the mRNA-optimized buffer (middle), and purified T7 RNAP (right). Each point represents one of the 60 T7 genes. The blue line indicates the linear regression fit (y = ax + b), and the dashed black line represents the identity line (y = x). Pearson’s correlation coefficient (r) and Spearman’s rank correlation coefficient (ρ) are reported in each panel. The 95% confidence intervals (CI) are shown in brackets; Pearson intervals were computed using a Fisher z-transformation, and Spearman intervals by bootstrap resampling (*n* = 1000). Source data are provided as a Source Data file.

To express entire genomes in CFS using the two selected buffers, we chose bacteriophage T7 as a model system because it is well characterized and its genome is extensively annotated^34^. Direct RNA nanopore sequencing was performed using an eight-step workflow (Fig. 4). First, T7 genomic DNA was extracted from infected *E. coli* by phenol-chloroform extraction to preserve genome integrity (Fig. 4a, step 1). The optimized cell-free reactions were then supplemented with the T7 genome. In parallel, reactions containing the reporter plasmid were monitored to determine the optimal RNA harvest time (Fig. 4a, step 2). Total RNA was subsequently isolated and purified by phenol–chloroform extraction (Fig. 4a, step 3). RNA was then polyadenylated to enable adapter ligation (Fig. 4a, step 4), and RNA integrity was assessed (Fig. 4a, step 5). Polyadenylated RNA libraries were ligated to sequencing adapters (Fig. 4a, step 6), loaded onto MinION flow cells, and sequenced in real time (Fig. 4a, step 7). The resulting long reads were aligned to reference sequences (Fig. 4a, step 8). The datasets were processed using a dedicated long-read RNA-seq analysis pipeline (“Methods”, Supplementary Fig. 6). Reads were concatenated per flow cell and aligned to a composite reference containing the T7 genome, the *E. coli* BL21(DE3) genome, and the *S. cerevisiae* ENO2 spike-in control. Base-level coverage profiles were then used to visualize genome-wide transcriptional patterns as coverage plots (Fig. 5, Supplementary Fig. 7). Gene expression differences between samples were normalized using DESeq2, and correlations across conditions were subsequently analyzed (Fig. 6, Supplementary Fig. 8). For comparison, the T7 genome was also expressed under two reference conditions: a minimal in vitro transcription reaction using purified T7 RNAP and its commercial buffer (Fig. 4a), and an in vivo infection of living *E. coli* cells (Fig. 4b).

### Genome expression profiles across systems of increasing complexity

In the minimalist system, only containing the purified T7 RNAP and its reaction buffer (NTPs, pH buffer, magnesium and DTT, see “Methods”), transcription was initiated from all T7-dependent promoters (Fig. 5a, bottom row). The very same phenol extracted T7 genome was used with the minimalist system as with cell-free expression systems (Fig. 4a). Coverage exhibited a 5′ bias, reflecting the non-equilibrium nature of a production-only system. Upon NTPs exhaustion, the reaction accumulates a mixture of full-length transcripts, partially elongated intermediates, and nascent RNAs. This results in a 5′-to-3′ coverage gradient, where 5′ regions are in all the transcripts, while 3′ regions are present exclusively in full-length molecules. This production-only property makes the minimalist system an ideal proxy for comparing T7 RNAP-specific promoter strengths in their native genomic context. The T7 promoters were revealed (purple lines annotated T7 in Fig. 5a) as well as the two T7 terminators (orange line in Fig. 5a). These results are consistent with the specificity of the T7 RNAP, as the *E. coli* promoters present no mRNA peaks (purple lines annotated *E. coli* in Fig. 5a). In addition, we compared promoter strength using normalized RNA-seq coverage using signal in the region +12 to +42 from the TSS to a local baseline, allowing us to isolate promoter escape from upstream effects. Our results broadly agree with previous rankings^35^ and complete them, allowing us to rank every T7-RNAP-dependent promoter of the T7 genome (Supplementary Table 1). Moreover, we observe that RNA coverage profiles revealed substantial variability among promoters sharing the identical consensus sequence (TAATACGACTCACTATAGGG), with relative strengths ranging from 0.34 to 0.71 depending on genomic location (Supplementary Fig. 9). This demonstrates that promoter strength is not solely sequence-determined but is strongly modulated by local genomic context even in the T7 phage genome. In CFS, transcripts from T7 RNAP-dependent promoters were detected as well (Fig. 5a, middle rows). Uniform transcript coverage reflected balanced transcription and degradation rates. In T7 phage biology, mRNA maturation is driven by RNase III-mediated processing^36^. Unlike the minimalist system, CFS restores T7 mRNA maturation, which is likely mediated by endogenous RNase III (Fig. 5b, bottom and middle rows). Finally, the read coverage from in vivo data was skewed toward the 3′ end of transcripts, indicating an imbalance between mRNA production and degradation (Fig. 5a, top row). It suggests that mRNA production and/or degradation differ from that observed in CFS. One hypothesis for the difference in degradation is the absence of membrane-associated proteins. Among the ribonucleases of *E. coli*, RNase E is membrane-associated^37,38^, and its absence in CFS^1^ could account for the observed differences.

### mRNA levels between in vivo and cell-free expression systems

mRNA coverage correlates well with in vivo measurements under both trade-off and mRNA-optimized buffer conditions, reflecting comparable promoter strengths and mRNA degradation rates (Fig. 6, middle and left panels). Gene expression in both the trade-off and mRNA-optimized buffers shows moderate to strong agreement with in vivo data, as indicated by positive linear regressions approaching the identity line. This pattern is consistent with previous observations in living cells, where promoter strength remains proportional across varying growth conditions^39^. Nevertheless, mRNA levels obtained with the minimalist system differ considerably from in vivo expression patterns, demonstrating that promoter strength alone is insufficient to predict mRNA steady-state levels (Fig. 6, right panel). Repeating our experiments revealed an interesting pattern in both CFS (optimized and trade-off) and in vivo conditions; the first 7 genes of the T7 genome showed high variability in expression between experiments. This shows that CFS captures the same variability as observed in vivo, which is not the case with the minimalist system (Supplementary Fig. 8). Eventually, our comparative analysis establishes cell-free systems as powerful platforms that bridge the gap between simplified reconstituted systems and complex in vivo environments, enabling systematic investigation of gene expression mechanisms.

## Discussion

By integrating active learning with high-throughput experimentation, we developed a machine learning-guided optimization pipeline that strongly increased mRNA yield in cell-free systems, enabling genome-wide transcriptomic profiling. This advancement overcomes the major limitation for cell-free transcriptomics and establishes an adaptable platform for studying gene expression across biological contexts.

Our active learning approach proved highly effective for exploring the vast combinatorial space of buffer compositions. Gaussian process regression outperformed other machine learning algorithms, providing accurate predictions with well-calibrated uncertainty estimates that guided efficient exploration. The buffer optimization revealed that Mg-glutamate, NTPs, 3-PGA, K-glutamate and DNA concentrations are critical for mRNA abundance, while spermidine, amino acids, and PEG-8000 showed no detectable effect on mRNA abundance within the tested ranges. Interestingly, most compositions maximizing mRNA yield lead to reduced protein production. Nevertheless, the identification of a trade-off buffer achieving both high mRNA and protein yields reveals underexplored regions of our parameter space where transcriptional and translational activities could be co-optimized. Exploiting the trade-off buffer could transform cell-free systems into powerful integrated multi-omics platforms, enabling simultaneous transcriptomic and proteomic profiling of gene expression.

Because active learning was conducted exclusively with T7 RNAP, the resulting buffer compositions are likely biased toward this transcription system and may not be fully optimal for transcription driven by the endogenous *E. coli* RNAP. This limitation is reflected in the substantially smaller improvement observed with *E. coli* RNAP (~4-fold for the mRNA-optimized buffer and ~2.5-fold for the trade-off buffer) compared to the ~20-fold and ~13-fold increase obtained with T7 RNAP. These differences suggest that both buffer conditions are at least partially specific to the T7 transcription machinery due to its structure or concentration in the cell-free reaction. Nevertheless, the active learning framework itself remains transferable. Transfer learning based on the T7 RNAP dataset could accelerate adaptation to endogenous polymerases, provided that the explored compositional space already captured conditions favorable to endogenous transcription. To test mRNA yield improvements based on the endogenous *E. coli* RNAP, we measured mRNA production from a plasmid carrying a strong constitutive *E. coli* promoter upstream of the *degfp* gene (Supplementary Fig. 10a). Both the optimized and trade-off buffers significantly increased mRNA yield compared to the reference buffer (four-fold 95% confidence interval of [3.48, 4.58] and 2.5-fold 95% confidence interval of [1.26, 2.7], respectively (Supplementary Fig. 10b), although the magnitude of improvement was lower than that observed with T7 RNA polymerase. Nevertheless, these results demonstrate that the identified buffer compositions also enhance mRNA levels produced with bacterial machinery, providing a basis for further species-specific refinement.

Our comparative analysis of T7 bacteriophage expression across four conditions (purified T7 RNA polymerase, mRNA-optimized cell-free buffer, trade-off buffer, and in vivo) reveals how different systems can capture distinct regulatory layers of gene expression. The minimalist purified polymerase system serves as a baseline to quantify promoter strength, producing transcripts without degradation and revealing genome location-dependent expression among promoters sharing the same T7-RNAP binding sequence. Cell-free systems restore mRNA maturation likely through endogenous RNase III activity. mRNA coverage correlates well with in vivo measurements under both trade-off and mRNA-optimized buffer conditions, demonstrating that relative expression levels are preserved despite global differences in buffer compositions. The distinct degradation patterns observed between cell-free and in vivo conditions reflect additional layers of regulation in living cells, potentially due to membrane-related function. While cell-free systems restore RNase III-mediated maturation, the 3′-biased coverage in vivo reveals the loss of specific mechanisms in cell-free systems. This progressive restoration of regulatory layers from transcription initiation with purified polymerase, to degradation processing in cell-free systems, to complete degradation machinery in living cells, provides a systematic framework for dissecting the molecular mechanisms controlling gene expression.

Eventually, our work establishes a foundation for cell-free transcriptomics of complete bacterial genomes using endogenous RNA polymerases. Unlike T7 RNA polymerase-driven expression, optimization for native or heterologously expressed bacterial transcriptional machinery would provide access to the full regulatory repertoire of any organism. This capability is particularly valuable for unculturable bacteria, which represent the vast majority of microbial diversity but remain inaccessible to conventional transcriptomic approaches. By bypassing the need for living cells, cell-free transcriptomics could reveal the transcriptional landscapes and regulatory networks of these organisms using only PCR-amplified and/or partially synthetically reconstructed genomic DNA. Furthermore, our approach extends naturally to non-model bacteriophages, enabling comparative studies of phage gene expression strategies and host-pathogen interactions under a controlled biochemical environment.

## Methods

### Strains and plasmids

Strain *E. coli* BL21 (DE3) (B F^–^ *ompT gal dcm lon hsdS*_*B*_(*r*_*B*_^–^*m*_*B*_^–^) λ (DE3 [*lacI lacUV5*-*T7p07 ind1 sam7 nin5*]) [*malB*^+^]_K-12_(λ^S^)) was used to prepare the lysate^40^. The plasmid PT7-deGFP-MGapt (4176 bp) was a gift from Richard Murray (Addgene plasmid # 67741; http://n2t.net/addgene:67741; RRID: Addgene_67741) and transformed into chemically competent *E. coli* DH5α (F^–^ *endA1 glnV44 thi-1 recA1 relA1 gyrA96 deoR nupG purB20* φ80d*lacZ*ΔM15 Δ(*lacZYA-argF*)U169, hsdR17(*r*_*K*_^–^*m*_*K*_^+^), λ^–^).

### Plasmid construction

The PT7-deGFP-MGapt plasmid was PCR amplified using the primers Forward: TTTTTTTTATCTGAAATACCGAATAACTAGCATAACCCCTTGGGG and Reverse:

CGGGATCCACTAGTCATTCAGCCGGATCTCCTAAGGCT. In parallel, the PNG1299 plasmid (Addgene plasmid #149709) was amplified using the primers Forward:

CCTTAGGAGATCCGGCTGAATGACTAGTGGATCCCGACTG and Reverse:

GGGGTTATGCTAGTTATTCGGTATTTCAGATAAAAAAAATCCT. The resulting PCR products were assembled using the Gibson Assembly method to generate the PGD2 construct.

To replace the PT7 promoter with the PR-OR1-OR2 promoter, PGD2 was subsequently PCR amplified using phosphorylated primers Forward:

[PHO]TTACCTCTGGCGGTGATAATGGTTGCATCCCTCTAGAAATAATTTTG and Reverse:

[PHO]AATTGTCAACACGCACGGTGTTAGCTCAATTTCGCGGGATCGAGATCT, yielding the PR-OR1-OR2-deGFP-MGapt construct used in Supplementary Fig. 10c.

### Plasmid extraction and purification

The plasmid PT7-deGFP-MGapt was extracted from an overnight LB culture of *E. coli* DH5α using the QIAGEN Plasmid Maxi Kit (Qiagen, ref 12165) following the manufacturer’s instructions. Since DNA preparation can significantly impact cell-free reactions^20,41^, we conducted all experiments using plasmid from the same initial stock. This stock was a combination of two batches prepared from 4 L and 3 L of the overnight LB culture with 100 µg/mL ampicillin, processed with 8 and 6 columns, respectively, and yielding approximately 4000 µg and 2400 µg of plasmid. The concentration of the stock was adjusted to 50 nM (135.5 ng/µL) and aliquoted into 450 µL each.

### Cell lysate preparation

Due to malachite green’s sensitivity to DTT, all DTT was removed from the buffers^42^. *E. coli* BL21 (DE3) strain was cultured in rich 2 x YT medium at 37 °C with 1 mM IPTG induction until the optical density reached 1.5–2.0. After two washes with DTT-free buffer S30A (14 mM Mg-glutamate, 60 mM K-glutamate, 50 mM Tris, pH 7.7), each pellet was resuspended in a specified volume of buffer S30A based on its mass (0.9 mL per gram). The resuspended cells were then divided into 1 mL aliquots in 1.5 mL Eppendorf tubes. These tubes were sonicated using a Sonics VCX 750 Vibra Cell with 20% amplitude, following the sequence: 40 s ON – 60 s OFF – 40 s ON – 60 s OFF – 40 s ON. The remaining steps include a 1h run-off reaction at 37 °C. The lysate was aliquoted into 2 mL Eppendorf tubes, with 1480 µL per tube, and stored at −70 °C. The final lysate stock was prepared by combining 7 independently tested batches, totaling 32 L of culture and yielding 62 mL of lysate.

### Cell-free reagents preparation

All products used and the final concentrations of our reagent stocks are listed in Supplementary Table 2. Given that reagent preparation can significantly impact cell-free reactions^41^, we ensured that all our reagents came from aliquots of the same initial batch. The only exceptions were NTPs and 3-PGA, as additional material was needed during the study. We observed no effect on our controls when the NTPs and 3-PGA were renewed (Supplementary Fig. 11).

To prepare the amino acid mixture, amino acids were grouped based on solubility and dissolved into three separate 36 × stock solutions: water-soluble (neutral), acid-soluble, and base-soluble. Each amino acid was weighed according to its molecular weight to reach a final concentration of 54 mM per compound in 20 mL of stock solution. Water-soluble amino acids (alanine, arginine, glycine, histidine, lysine, proline, serine, threonine, and valine) were dissolved directly in ultrapure water. Acid-soluble amino acids (aspartic acid, asparagine, cystine, glutamine, glutamic acid, leucine, methionine, tryptophan, and tyrosine) were solubilized in 1 M HCl. Base-soluble amino acids (isoleucine and phenylalanine) were solubilized in 1 M KOH. Prior to use in cell-free experiments, the three stock solutions were mixed in equal volumes to yield a complete amino acid mixture.

### Plasmid-based cell-free reaction

Reactions took place in 10.5 μL volumes at 30 °C in a 384-well plate for 15 h. To assemble the cell-free reactions, we used the Echo 650 liquid handler (Beckman, USA) to sequentially add a fixed amount of HEPES (50 mM), the variable buffer component, and finally, a fixed amount of malachite green (0.010 mM). The volume was then manually adjusted to 10.5 µL by adding a fixed amount of lysate (3.5 µL) and the appropriate volume of water using an automatic multi-channel pipette. The plate was immediately centrifuged at 1000 *g* for 1 min at 4 °C, sealed, and incubated in a plate reader preheated to 30 °C.

### Fluorescence measurements

We used a BioTek Synergy HTX plate reader (Agilent) to measure fluorescence in clear, flat-bottom 384-well plates (Greiner ref #781185). The plate was incubated for 15 h at 30 °C, with fluorescence measurements taken every 6 min and 7 s. The malachite green aptamer fluorescence was measured with excitation at 590 ± 20 nm and emission at 635 ± 20 nm, using a gain of 100 for plates 1–7 and a gain of 85 from plate 8 onward. deGFP fluorescence was measured with excitation at 425 ± 20 nm, emission at 510 ± 20 nm, and a gain of 55. Fluorescence was detected from the top of the 384-well plate, which was sealed with clear sealing tape (Revivity ex. PerkinElmer #6050185 for plate 0 and ThermoFisher #232701 from plate 1 onward).

### Definition of the reference buffer and combinatorial space

For *E. coli* lysate-based cell-free systems, a widely used method is the protocol established by Sun et al.^4^. In 2020, Borkowski et al. systematically optimized this buffer for protein production and identified five components that had minimal impact on protein yield^20^. In the present study, we adopted the Sun et al. buffer as a reference, omitting the non-essential components identified by Borkowski et al., resulting in the following composition: 4 mM Mg-glutamate, 120 mM K-glutamate, 1.5 mM amino acids, 1 mM spermidine, 30 mM 3-PGA, 1.5 mM of ATP and GTP, 0.9 mM of UTP and CTP, 2% PEG-8000, 50 mM HEPES. For the original combinatorial space, we defined six concentrations per component, anchoring the fifth at the reference level to enable exploration of four lower and one higher concentration.

### Data analysis and normalization

The script “From_biotek_data_to_active_learning_vf.py” calculates a normalized value from the time-course malachite green aptamer and deGFP fluorescence data obtained from the plate reader, enabling the comparison of mRNA and protein concentrations between buffers relative to a reference buffer. Briefly, the time-course fluorescence data were corrected well-by-well by subtracting the background fluorescence of the plastic, which was measured by reading the empty plate in the plate reader prior to filling it. For each well, the maximum fluorescence value was selected to calculate the yield as following:

1 $${{Yield}}_{{Buffer\; x}}=\left(\frac{{{Fluo}}_{max }({Buffer}\,x)-{{Fluo}}_{max }({Ref}-)}{{{Fluo}}_{max }({Ref}+)-{{Fluo}}_{max }({Ref}-)}\right)$$

Where:

${{Fluo}}_{max }({Buffer\; x})$: For malachite green fluorescence, this is the maximum fluorescence value over time in each buffer composition. For GFP fluorescence data, it is the value after 15 h, corresponding to the maximum fluorescence value in each buffer composition.

${{Fluo}}_{max }({Ref}+)$: The median of the maximum fluorescence value (malachite green or deGFP) in the reference buffer containing 10 nM of DNA.

${{Fluo}}_{max }({Ref}-)$: The median of the maximum fluorescence value (malachite green or deGFP) in the reference buffer without DNA, representing the autofluorescence of the cell-free reaction, which is primarily due to the lysate autofluorescence.

Each yield represents the median of multiple replicates (3 for plates 0 to 3, and 6 for plates 4 to 10).

Starting from plate 7, the active learning process successfully identified buffer compositions that substantially increased mRNA production, as reflected by higher malachite green fluorescence. This increase required adjusting the gain on the plate reader, which made the original reference buffer with DNA (*P0_Ref*+) indistinguishable from background noise. To correct for this, we used an alternative reference buffer (*corr_buffer*) that was previously measured in plates with the old gain, and recalculated yields as:

2 $${{Yield}}_{{Buffer}\,x}=\left(\frac{{{Fluo}}_{max }({Buffer}\,x)-{{Fluo}}_{max }({Ref}-)}{{{Fluo}}_{max }({corr}{{\_}}\,{buffer})-{{Fluo}}_{max }({Ref}-)}\right) * {scalingfactor}$$

Where the $${scaling}\,{factor}=\frac{{{Fluo}}_{max }\left({{\rm{corr}}}\_{{\rm{buffer}}}, {{\rm{corr}}}\_{{\rm{plate}}}\right)\,-\,{{Fluo}}_{max }\left({Ref}-, {{\rm{corr}}}\_{{\rm{plate}}}\right)}{{{Fluo}}_{max }\left({Ref}+,{{\rm{corr}}}\_{{\rm{plate}}}\right)\,-\,{{Fluo}}_{max }\left({Ref}-,{{\rm{corr}}}\_{{\rm{plate}}}\right)}$$

This correction can be computed using a scaling factor derived from:

- any buffer that was measured both in plate 8 (after the gain change) and in at least one plate before the gain change (e.g., *corr_buffer* = *P5_Buffer 21*),
- and any plate prior to the gain change in which this buffer was measured (defined as *corr_plate*; e.g., *P5_Buffer 21* in plate 5, 6, or 7).

To select the optimal correction, we chose *P5_Buffer 21* as the *corr_buffer* because, among the control buffers retested in multiple plates, it was the first to remain clearly distinguishable from background at both gain settings. For the *corr_plate*, we compared the buffer rankings computed using (i) the scaling factor applied to the yield and (ii) a simplified fluorescence ratio metric (*ratio_fluo*) that does not subtract the autofluorescence to allow consistent comparison across plates 5, 6, 7, 8, 9 and 10 at both gain settings:

3 $${{Ratio}{{\_}}\,{fluo}}_{{Buffer}\,x}=\frac{{{Fluo}}_{max }({Bufferx})}{{{Fluo}}_{max }(P5{{\_}}\,{Buffer}21)}$$

This comparison was performed for all buffers in plates 5–10. The best correlation between the yield-based ranks and *ratio_fluo*-based ranks was observed when using *P5_Buffer 21* in plate 7 as the reference (Supplementary Fig. 12). Using this correction, the final yield calculation for plates 8, 9 and 10 was:

4 $${{Yield}}_{{Buffer}\,x}=\left(\frac{{{Fluo}}_{max }({Buffer}\,x)-{{Fluo}}_{max }({Ref}-)}{{{Fluo}}_{max }(P5{{\_}}\,{Buffer}\,21)-{{Fluo}}_{max }({Ref}-)}\right) * 6.95$$

where 6.95 is the scaling factor determined from *P5_Buffer 21* in plate 7.

The composition of P5_Buffer 21 is: 2.01 mM Mg-glutamate, 40 mM K-glutamate, 2.25 mM amino acids, 0.50 mM spermidine, 45 mM 3-PGA, 3.74 mM NTPs, 2.49% PEG-8000, 50 mM HEPES and 8.3 nM DNA.

To ensure that our normalization was sufficient for comparing data between plates, we measured a collection of control buffers on each plate and compared them across plates. The list of controls was iteratively updated over time to include buffers with increasingly higher fluorescence levels, reflecting the progression of active learning towards the optimal buffers. These controls validated that our normalization effectively corrected plate-to-plate variations, such as the change in plate sealer between plates 0 and 1 and the change in gain between plates 7 and 8 (Supplementary Fig. 13).

### Initialization of the active learning loop by Latin Hypercube sampling

To initiate the active learning loop, the initial 100 experiments were selected using Latin Hypercube Sampling (LHS) to ensure good coverage of the search space. LHS assumes the independence of input variables (X). For each variable, the cumulative distribution function (based on a uniform distribution in our case) is divided into *n* equally sized partitions. A random value is then selected from each partition. These sampled values are combined across all variables to form *n* unique samples of X. One advantage of LHS arises when the output (Y, yield) is dominated by only a few components of X (cell-free mix components). This method ensures that these components are fully represented in a stratified manner, regardless of which components turn out to be important^43^. LHS is implemented by the SAMPLER module of the *icfree-ml* package (https://github.com/brsynth/icfree-ml), further described in Khalil et al.^44^.

### Active learning modeling

The script “active_learning_bayesian_style_vf.py” generates a list of buffer compositions to test in the next plate according to active learning. Yield optimization was performed using a Bayesian optimization active learning approach, with a Gaussian Process (GP) model to probabilistically estimate the relationship between component concentrations and yield. In each active learning cycle, the model provided a set of experiments in CSV format for execution using an Echo liquid handler.

#### Choice of the surrogate function

To select the more accurate surrogate function, we evaluated the predictive performance of four common machine learning algorithms: Multilayer Perceptron (MLP), Random Forest (RF), Gaussian Process (GP), and XGBoost. The MLP is a widely used feed-forward neural network in which signals propagate from input to output without recurrent connections. While highly flexible, it requires careful tuning of architecture and hyperparameters to avoid overfitting. RF is a bagging-based ensemble method that combines multiple decision trees, each trained on a random subset of the data, to improve accuracy and generalization. XGBoost is an optimized gradient-boosting framework that iteratively builds decision trees while including a regularization term to prevent overfitting and enhance generalization. GP regression is a non-parametric Bayesian method that assumes outputs for any finite set of inputs are jointly Gaussian distributed. It provides both accurate predictions and well-calibrated uncertainty estimates, making it particularly suitable for optimization tasks such as Bayesian optimization. While MLP and XGBoost have been successfully applied to similar tasks^20,21^, RF and GP tend to perform more robustly on limited datasets. To compare these models, we applied repeated random sub-sampling validation, randomly splitting the dataset into 80% training and 20% testing subsets over 50 iterations, and evaluated performance using the coefficient of determination (R²), which quantifies the proportion of variance in the observed data explained by the model. R² ranges from negative infinity to 1, where 1 indicates a perfect fit, 0 corresponds to predicting the mean, and negative values indicate worse-than-mean performance. All models (MLP, RF, XGBoost, and GP) were systematically tuned during the experiments. For each training subset, a grid search with five-fold cross-validation was performed, and the best hyperparameters were then retrained on the full training set. The MLP was tuned for different hidden layer sizes (32, 64), (64), (32), and (10) to keep the network small and reduce overfitting. Random Forest was tuned for the number of estimators (10, 50) and maximum tree depth (5, 10), while XGBoost was tuned for the learning rate (0.01, 0.1), the number of boosting rounds (100, 500, 700), and maximum tree depth (3, 5, 7). Gaussian Processes were tuned for different kernel combinations (White, RBF, and Matérn) and for assumed noise variance (0.01, 0.05, 0.1). All models were trained and evaluated on identical data splits at each repetition to ensure a fair comparison. We observed that GP consistently outperformed the other algorithms, exhibiting the highest accuracy and reliability (Supplementary Fig. 1a)

#### Choice of the acquisition function

Optimization was driven by the Upper Confidence Bound (UCB) acquisition function, the expected improvement (EI) function, or the probability of improvement (PI) function, depending on the iteration. During the early cycles within a given combinatorial space, we used UCB with a manually adjusted exploration–exploitation balance to efficiently target high-potential regions of the search space. After two loops without noticeable improvement, we shifted the balance toward exploitation, using EI or PI, a choice guided by preliminary simulations showing that most genuine improvements occur within the first two to four loops (Supplementary Fig. 1b). Continuing exploration beyond this point tended to produce redundant evaluations, making exploitation the most practical strategy to accelerate convergence. Upon each redefinition of the combinatorial space, the acquisition function was reset to maintain balance, and subsequent iterations again favored exploitation when promising regions were rapidly identified. We compared two strategies on plate 3: half of the compositions were selected using a purely exploitative approach (EI), and half using a purely exploratory approach (UCB).

### Automated workflow

Modeling, experimental plate design, and translation into Echo instructions were automated using the LEARNER, DESIGNER, and INSTRUCTOR modules from the *icfree-ml* package (https://github.com/brsynth/icfree-ml), further described in Khalil et al.^44^.

### Amplification of T7 phage

A fresh colony of *E. coli* MG1655 grown on LB agar was used to inoculate a 5 mL overnight culture in Lennox rich medium, incubated at 37 °C with shaking at 200 rpm. The next day, the culture was diluted 1:100 into 5 mL of fresh Lennox medium supplemented with 10 mM MgSO₄ and grown to an optical density at 600 nm (OD₆₀₀) of 0.2. Two 1 mL aliquots were collected; one was infected with T7 phage at a multiplicity of infection (MOI) of 10^−^^4^ (i.e., 2 × 10^−^^4^ pfu per cell). After 10 min of adsorption at room temperature, both aliquots were diluted into separate 250 mL Erlenmeyer flasks containing 50 mL of prewarmed Lennox supplemented with 10 mM MgSO₄. The uninfected culture served as a parallel control to monitor OD₆₀₀, allowing an estimate of bacterial density in the infected culture during lysis. Once complete lysis was observed, the infected culture was centrifuged at 4000 *g* for 10 min, and the supernatant was filtered through a 0.2 µm membrane. The resulting lysate was stored at 4 °C and titrated.

### Titration of T7 phage stock

A single colony of *E. coli* MG1655 was used to inoculate 3 mL of rich 2 × YTP medium and grown overnight at 37 °C with shaking at 200 rpm. The next day, 2 mL of fresh 2 × YTP medium supplemented with 10 mM MgSO₄ was inoculated with a 1:10 dilution of the overnight culture and incubated under the same conditions until the exponential phase. In parallel, serial 1:100 dilutions of the T7 phage stock (to be quantified) were prepared in 1 mL of SM buffer (50 mM Tris-HCl pH 7.5, 100 mM NaCl, 10 mM MgSO₄) to generate 10^−^^1^, 10^−^^3^, 10^−^^5^, 10^−^^7^, and 10^−^^9^ dilutions.

A soft LB-agar medium (7% agar, 10 mM MgSO₄) was melted and kept at 50 °C. For each phage dilution, 100 µL of the exponential-phase bacterial culture was spotted on one side of an empty Petri dish, and 100 µL of the corresponding phage dilution was spotted on the opposite side. The melted LB-agar was then poured into the plate, allowing the two drops to merge. Plates were gently rocked back and forth to ensure homogeneous distribution and left at room temperature until the agar solidified. After overnight incubation at 37 °C, the phage titer was estimated by counting lysis plaques and accounting for the dilution factor, expressed in plaque-forming units per milliliter (pfu/mL).

### Purification of T7 phage genome

Phage T7 was isolated using 50 ml of *E. coli* MG1655 growing in Lennox medium (Sigma-Aldrich, L9234). Then, we centrifuged at 5000 *g* for 15 min and filtered (0.2 µM) the lysed bacterial culture to remove debris. Phages were then concentrated by adding NaCl (to 0.5 M) and PEG-8000 (to 10%) and incubating overnight at 4 °C. After centrifugation at 5000 *g* for 15 min, the pellet was resuspended in SM buffer, and RNase A and DNase I (1 U/µl) are added to remove RNA and digest free DNA. Following a 30-min incubation, DNase I was inactivated with EDTA (0.5 M). Phage DNA was then extracted using phenol-chloroform and precipitated with potassium acetate and ethanol. The DNA was washed, resuspended in water, and quantified using a Qubit fluorometer (Invitrogen, #Q33226).

### Genome-based expression and RNA purification in the minimalist system

The T7 genome was expressed using purified T7 RNA polymerase (TranscriptAid T7 High Yield Transcription Kit, Thermo Fisher #K0441) in a 20 µL reaction. After a 2-h incubation at 37 °C, the RNA was treated with DNase I (1 U/µl) for 15 min at 37 °C, followed by 2 µL 0.5 M EDTA (pH 8.0) and incubation at 65 °C for 10 min to stop the reaction. RNA was then purified using the Monarch RNA Cleanup Kit (NEB T2050), polyadenylated (see section Polyadenylation of RNA), and sequenced (see section Long-read direct RNA sequencing with Oxford Nanopore Technology).

### Genome-based expression and RNA purification from cell-free reactions

The genome-based cell-free reactions were performed in the same way as the plasmid-based cell-free reactions, except that the DNA was not sent by the ECHO liquid acoustic handler but added manually just before the lysate. To prevent digestion of the linear T7 genome in cell-free, a Gibson reaction was performed, taking advantage of the 160 bp repeated sequence naturally present in the T7 genome (NEBuilder HiFi DNA Assembly Master Mix, NEB #E2621). DNA was dialyzed for 30 min using 0.025 µM membrane (Millipore #VSWP01300) before use. RNA extraction from cell-free reactions was performed after 3 h of reaction, which corresponds to the time to reach peak RNA concentration in the mRNA-optimized buffer (P10_Buffer 9).

RNA was purified from the cell-free reaction by phenol/chloroform extraction. The samples were extracted twice with an equal volume of acid phenol/chloroform/isoamyl alcohol (25:24:1, [pH 4.5]) and once with chloroform/isoamyl alcohol (24:1). After adding 1/10 volume of 3 M sodium acetate (pH 5.2), RNA was precipitated with isopropanol, washed with 70% ethanol and dissolved in 100 μL of RNase-free water. The samples were then DNase-treated using the RNase-Free DNase Set (Qiagen, #79256) and purified using the RNA Clean-Up and Concentration Micro Kit (Norgen #23600). The RNA concentration was measured using a NanoDrop spectrophotometer (ND-1000 LabTech); the quality of the RNA preparations was assessed by means of the Agilent 2100 Bioanalyzer according to the manufacturer’s instructions.

### Infection and RNA purification for in vivo experiment

An overnight culture of *E. coli* MG1655 was prepared in 2 mL of rich 2 × YTP medium supplemented with 10 mM MgSO₄. The culture was diluted 1:400 into 150 mL of fresh 2 × YTP medium and incubated at 37 °C with shaking at 200 rpm until the optical density at 600 nm (OD₆₀₀) reached 0.5. A second 1:400 dilution was then used to inoculate 150 mL of 2 × YTP for the main culture. Upon reaching OD₆₀₀ = 0.3 (corresponding to approximately 1.5 × 10^8^ cells/mL), the culture was split in two, and one half was infected with T7 phage at a multiplicity of infection (MOI) of 1. Ten minutes post-infection—within the estimated duration of the T7 lytic cycle (5–12 min)— cells were harvested by centrifugation (3 min, 8000 × *g*, 4 °C), and the pellet was flash-frozen in liquid nitrogen and stored at −70 °C.

The next day, total RNA was isolated. Briefly, bacterial cultures were mixed with one-half volume of frozen killing buffer (20 mM Tris-HCl pH 7.5; 5 mM MgCl₂; 20 mM NaN₃), and cells were harvested by centrifugation at 4 °C for 3 min. After discarding the supernatant, cell pellets were flash-frozen in liquid nitrogen and stored at –70 °C. For mechanical lysis, pellets were resuspended in 200 µL of ice-cold killing buffer and immediately transferred to a precooled Teflon disruption vessel containing liquid nitrogen. Samples were disrupted using a Mikro-Dismembrator S (Sartorius) for 2 min at 2600 rpm. The resulting frozen powder was resuspended by pipetting in 4 mL of lysis buffer prewarmed to 50 °C (4 M guanidine thiocyanate, 25 mM sodium acetate pH 5.2, 0.5% [wt/vol] N-laurylsarcosinate). Aliquots of 1 mL were transferred to microcentrifuge tubes and immediately flash-frozen in liquid nitrogen. Total RNA was purified by phenol–chloroform extraction, as described in the preceding section.

### Polyadenylation of RNA

For direct RNA sequencing with Oxford Nanopore Technology (ONT), polyadenylation was performed following the ONT protocol (v4, March 2023) using *E. coli* Poly(A), Polymerase (NEB: M0276L). This version of the ONT protocol is provided as “3-poly-rna-ecoli-pap.pdf” and is available for download at: https://nanoporetech.com/document/extraction-method/3-poly-rna-ecoli-pap?format=versions. Briefly, RNA was incubated at 37 °C for 1 min with 5 U of Polya, Polymerase and 1 mM ATP, then the reaction was halted with EDTA. Polyadenylated RNA was purified using beads (RNAClean XP Kit).

### Long-read direct RNA sequencing with Oxford Nanopore Technology

Library preparation was performed using the Direct RNA Sequencing Kit (SQK-RNA004, Oxford Nanopore Technologies, ONT) and sequenced on Flow Cell RNA (FLO-MIN004RA, ONT) using a MinION Mk1B device controlled by MinKNOW v24.06.5. The ONT protocol used in this study was an early access version, which is provided as “direct-rna-sequencing-sqk-rna004-DRS_9195_v4_revB_20Sep2023-minion.pdf” and is available for download at: https://community.nanoporetech.com/attachments/11348/download. RNA Control Strand (RNACS), corresponding to the *S. cerevisiae* ENO2 gene (YHR174W), was added to all samples at a defined concentration as an external spike-in, following protocol recommendations.

### RNA-seq data analysis

RNA-seq data analysis was performed using the Migale bioinformatics facility. The complete analysis workflow is presented in Supplementary Fig. 6, including the names and versions of all bioinformatics tools used, the custom Python scripts developed for this study, and the intermediate files available in the accompanying data repository (10.57745/BHTM60). Code snippets and usage details are provided in the repository report.

#### Data preparation

Only reads from the “pass” folder generated by MinKNOW were used, corresponding to reads passing the initial quality threshold (Q score > 8). Raw direct RNA sequencing reads were concatenated into a single FASTQ file per flow cell. Raw RNA reads are available at the NCBI SRA (ID: PRJNA1217614). To ensure compatibility with standard DNA-based bioinformatics tools, uracil (U) bases were converted to thymine (T).

#### Quality control and reads cleaning

Read quality was assessed using seqkit^45^, FastQC (Simon A., https://www.bioinformatics.babraham.ac.uk/projects/fastqc/ (2010)), MultiQC^46^, NanoStat^47^ and NanoPlot^47^, allowing evaluation of read length distributions, GC content, quality score distributions, and overall sequencing performance. Reads were filtered using Chopper^47^, a tool optimized for long-read data, retaining only those with GC content between 30–65% and lengths between 100 and 40,000 bases, thereby removing aberrant reads and sequencing artifacts. The total number of reads obtained for each flow cell is reported in Supplementary Fig. 14.

#### Read mapping

As expected, reads mapped to three reference sequences: (i) the T7 genome, (ii) the *E. coli* BL21(DE3) genome, and (iii) the *S. cerevisiae* ENO2 gene (YHR174W), used as an external spike-in control. Although the lysate is expected to be free of *E. coli* genomic DNA and RNA, degradation fragments from ribosomal RNA (16S and 23S rRNA) may generate reads mapping to the *E. coli* genome. The three references were concatenated into a composite reference genome (3refs.fasta). To prevent redundancy, the T7 genes were mapped to the *E. coli* genome using BWA^48^ and masked using BEDTools^49^. Mapping with BWA identified only the gene encoding T7 RNAP as redundant in the *E. coli* genome; therefore, only this gene was masked using BEDTools. Reads were aligned to this composite reference using minimap2, with secondary and supplementary alignments disabled^50^.

#### Reference-specific read quantification

Using alignment flags in the resulting BAM files, the proportion of reads mapping to each of the three references was computed using SAMtools^51^ (Supplementary Fig. 14). Although RNACS was included as a spike-in following the Oxford Nanopore Technologies Direct RNA Sequencing Kit recommendations, retrospective analysis revealed that it was not stable over time: measurements of RNACS-only tubes using Qubit showed variable RNA concentrations depending on storage duration. For this reason, spike-in normalization was not applied. This did not affect the analyses presented here, as all comparisons were based on relative expression patterns and coverage profiles rather than absolute RNA quantities.

#### Coverage analysis

Base-level coverage was extracted from BAM files using SAMtools depth^51^, producing three-column text files (chromosome, position, depth) named “ID_vs_REF_coverage.txt”^51^.

#### TPM quantification

Gene expression levels were estimated from the BAM alignments using BEDTools^29^. Output included transcript-level TPM values stored in “ID_vs_REF.tpm.tsv”, calculated as follows:

$${{TPM}}_{i}=\frac{{{FPKM}}_{i}}{{\sum }_{j}{{FPKM}}_{j}} * {10}^{6}$$ with $${{FPKM}}_{i}=\frac{{{counts}}_{i} * {10}^{9}}{{{length}}_{i} * {total\_counts}}$$

#### DESeq2 quantification

To compare gene expression between samples, a custom R script (DESEQ2norm.R) was used to normalize counts with DESeq2. Values were stored in files named “ID_vs_REF_coverage_TPM_by_gene.tsv.DESEQ2.tsv”.

#### Data integration and visualization

Python scripts were used to integrate and visualize expression data. The script “create_tables.py” merged per-base coverage with TPM values, producing two output files: “ID_vs_REF_coverage_TPM_by_position.tsv” and “ID_vs_REF_coverage_TPM_by_gene.tsv”, summarizing base-level and gene-level data, respectively. The script “plot_coverage.py” was used to generate coverage plots for each sample (Fig. 5, Supplementary Fig. 7). The script “plot_correlation_DESEQ2.py” was used to compare DESeq2-normalized expression values between flow cells (Fig. 6, Supplementary Fig. 8).

### Reproducibility

All figures presented in the main text correspond to a single sequencing run per sample. To assess reproducibility, biological replicates were independently prepared for each condition, encompassing the full workflow from RNA expression to sequencing (Fig. 4). Genome-wide coverage plots for these replicates are presented in Supplementary Fig. 7. Gene-level expression reproducibility is illustrated in Supplementary Fig. 8.

### Reporting summary

Further information on research design is available in the Nature Portfolio Reporting Summary linked to this article.

## Supplementary information

Supplementary Information

Description of Additional Supplementary Files

Supplementary Data 1

Reporting Summary

Transparent Peer Review file

## Source data

Source Data

## Data Availability

All data related to buffer optimization by active learning are provided in Supplementary Data 1 (csv file in supporting information) and on Zenodo (10.5281/zenodo.14980239). Raw RNA reads are available at NCBI SRA (ID: PRJNA1217614 https://www.ncbi.nlm.nih.gov/bioproject/?term=PRJNA1217614). Source data are provided with this paper.

## Code availability

All scripts were developed in Python (version 3.9). Scripts. The code used to develop the model, perform the analyses and generate results in this study is publicly available and has been deposited in Zenodo at 10.5281/zenodo.14980239 under MIT license. The specific version of the code associated with this publication is archived in Zenodo and is accessible via 10.5281/zenodo.14980239. The script for generating Echo instructions and performing Latin Hypercube Sampling (LHS) is included in the icfree-ml package (https://github.com/brsynth/icfree-ml^52^), further described in Khalil et al.^44^. Additionally, scripts and data for transcriptomics analyses can be found on Recherche Data Gouv (10.57745/BHTM60).
