# Supplementary material for: Active-learning-guided optimization of cell-free systems for genome-wide transcriptomic profiling reveals progressive layers of regulation
Source: Nat Commun. 2026 Jun 27;17:8065. doi: 10.1038/s41467-026-74559-y (PMC13454534; doi:10.1038/s41467-026-74559-y)
Supplement: Supplementary file 1 [file 41467_2026_74559_MOESM1_ESM.pdf]

Supplementary material

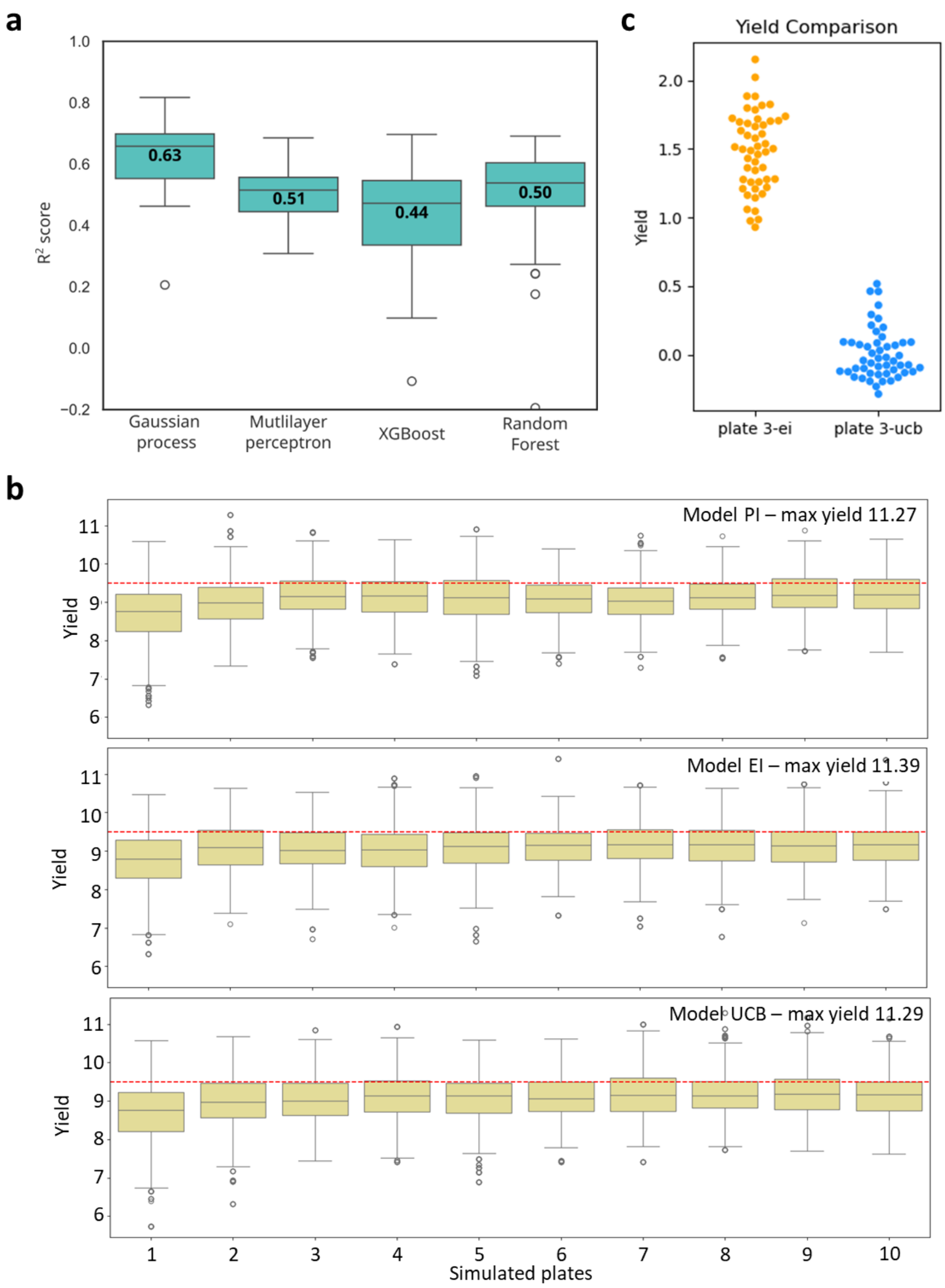

Supplementary Fig. 1 | Active learning model evaluation and acquisition strategy analysis. **a,** Distribution of R^2 values over 50 independent runs for four regression models—Multilayer Perceptron (MLP), Random Forest (RF), XGBoost, and Gaussian Process (GP). At each run, the dataset was randomly split into 80% training and 20% testing subsets. Model performance was evaluated using the coefficient of determination (R^2). The center line denotes the median, boxes indicate the interquartile range, and points outside the boxes represent outliers; mean R^2 values are indicated in bold. All models were systematically tuned using grid search with 5-fold cross-validation on the training data and retrained with optimal hyperparameters before evaluation. Gaussian Processes consistently achieved the highest accuracy and robustness across runs. **b,** Simulations of three acquisition functions—expected improvement (EI), probability of improvement (PI), and upper confidence bound (UCB)—showing that most performance gains occur within the first two to four active learning iterations. **c,** Swarm plot of yields obtained under two optimization strategies tested on plate 3, in which half of the compositions were selected by a purely exploitative strategy (EI) and the other half by a purely exploratory strategy (UCB). Exploratory samples did not yield improvements beyond model predictions, indicating that no higher-yield optima were present in underexplored regions of the parameter space. Source data are provided as a Source Data file.

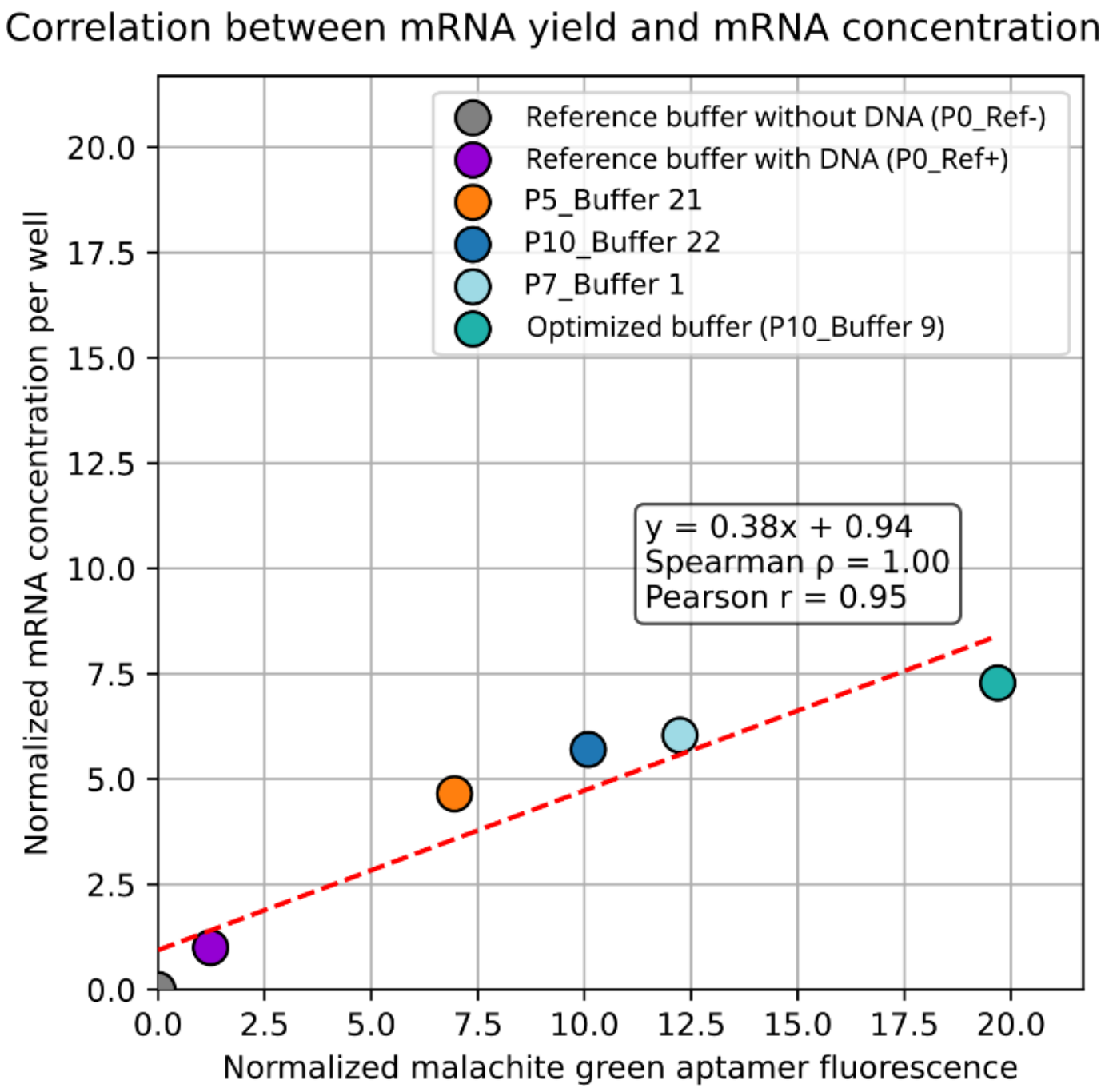

Supplementary Fig. 2 | Correlation of mRNA yield with purified mRNA concentration for representative buffers.

Cell-free reactions were performed with the indicated buffers and the reporter plasmid PLW1 (6 replicates per condition (Methods: plasmid-based cell-free reactions). Malachite green aptamer fluorescence was measured and normalized (Methods: Fluorescence measurements; Data analysis and normalization). At the maximum fluorescence, mRNA was purified for each condition (Methods: Genome-based expression and RNA purification from cell-free reactions), and total RNA concentration was quantified using Bioanalyzer. Purified mRNA concentrations were normalized using the same yield formula to enable direct comparison with the fluorescence-based yield. The correlation shows a slope of 0.38, likely reflecting material loss during RNA purification. The mRNA-optimized buffer enabled purification of ~7.5× more RNA than the reference buffer, corresponding to the same order of magnitude of improvement as measured by the fluorescence-based yield (20-fold; 95% CI [16.45, 21.33]). Source data are provided as a Source Data file.

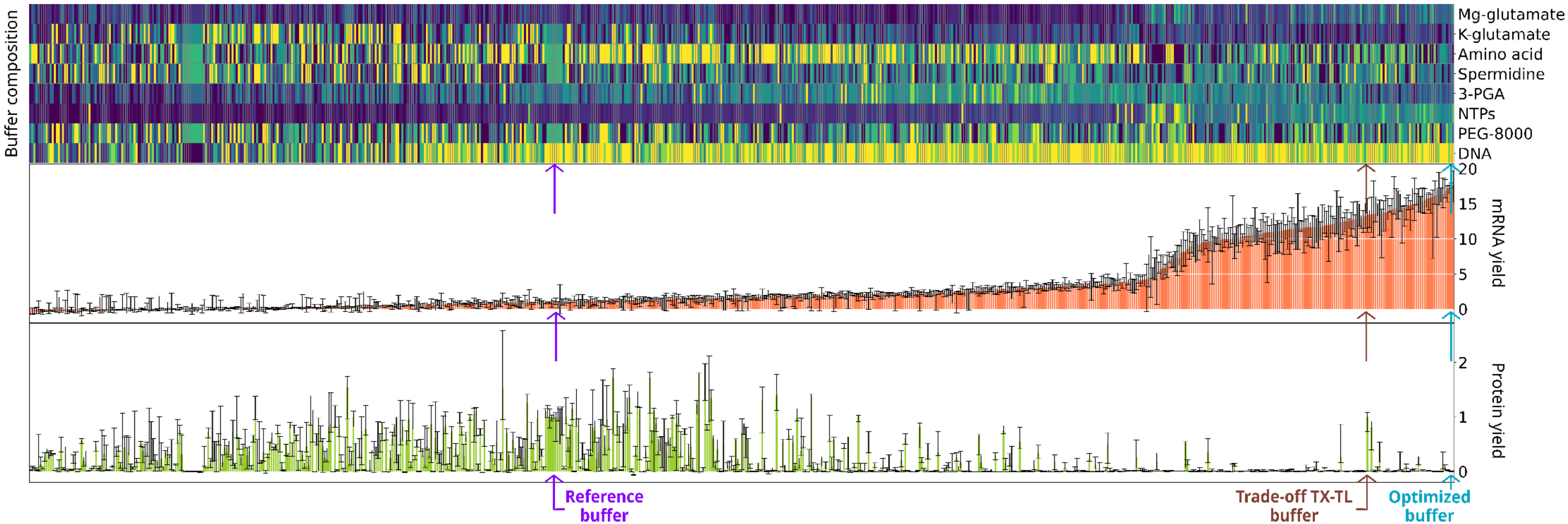

Supplementary Fig. 3 | Buffer composition, mRNA yield and protein yield ranked by mRNA yield. Buffer optimization across 10 rounds of active learning. The heatmap gives the composition of the tested buffers, with the minimum and maximum concentrations respectively in blue and yellow. The median of mRNA (coral barplot) and protein (green barplot) yield is indicated for each tested buffer. Error bars represent the 95% confidence interval across replicates (n=3 for plates 0-3; n=6 for plates 4-10). The 3 buffer conditions defined in Fig. 3b are highlighted (reference with DNA, trade-off and mRNA-optimized buffers, in purple, brown and blue respectively). Source data are provided as a Source Data file.

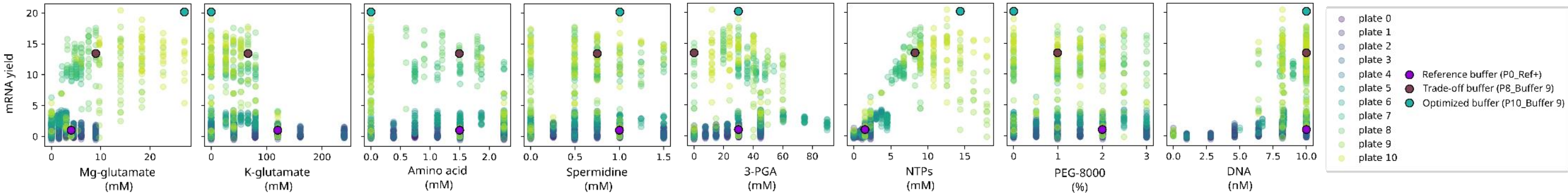

Supplementary Fig. 4 | Effect of compound concentration on mRNA yield during active learning optimization. Scatter plots showing the relationship between mRNA yield and the concentration of each compound. Each subplot corresponds to one of the eight compounds varied during optimization. Each point represents a unique buffer composition tested as suggested by the active learning model. Point color reflects the optimization trajectory across experimental rounds (plates), illustrating how the model progressively favored increasing concentrations of Mg-glutamate, NTPs, and DNA, while converging toward lower concentrations of K-glutamate. The 3 buffer conditions defined in Fig. 3b are highlighted (reference, trade-off and mRNA-optimized buffers). Source data are provided as a Source Data file.

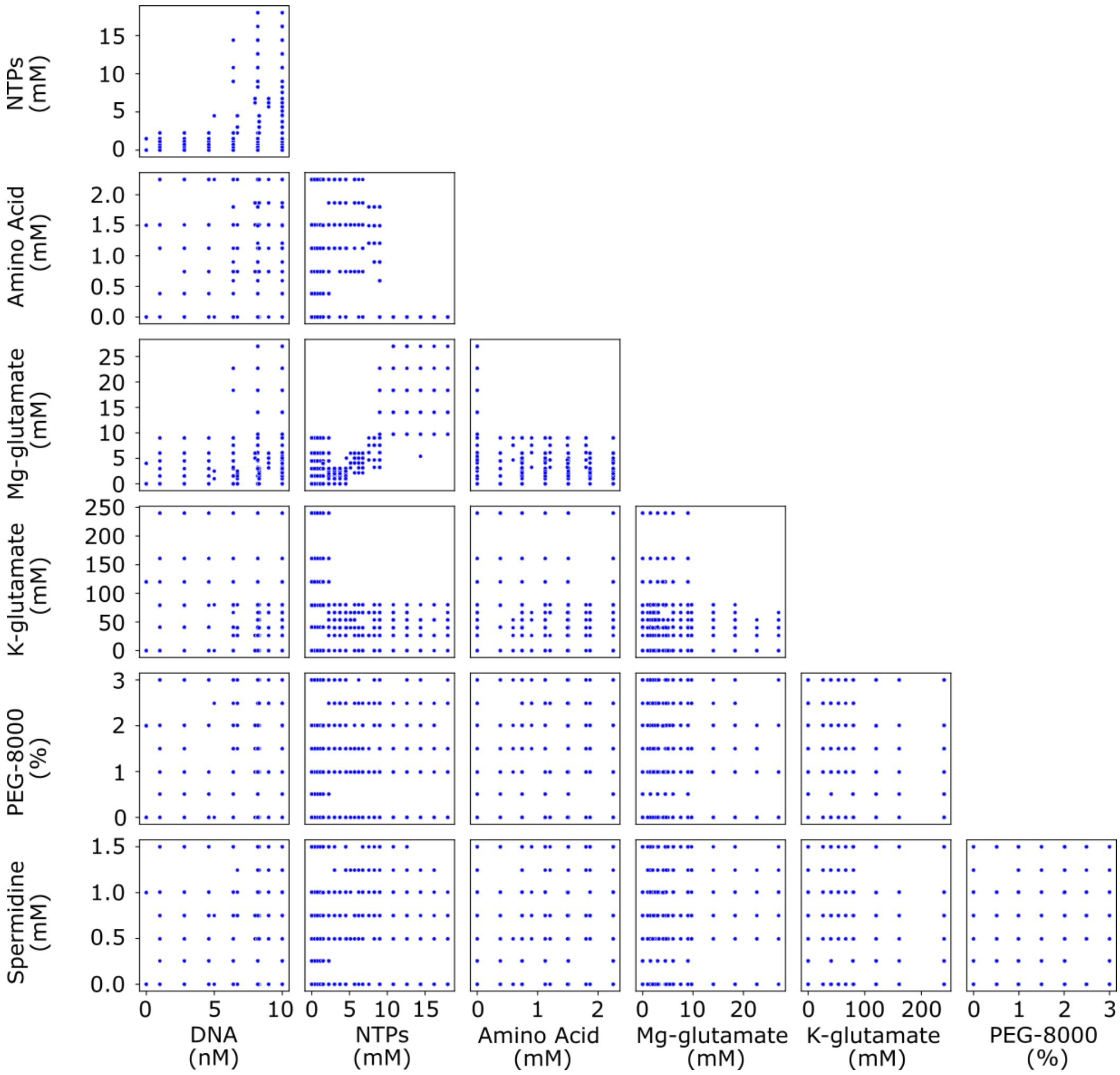

Supplementary Fig. 5 | Pairwise scatter plot of the buffer compounds concentrations sampled during our exploration. The subplot represents the pairwise relationship between two compounds, with individual blue dots corresponding to specific tested combinations. The plot reveals regions of the combinatorial space that were densely explored, as well as areas that remain under-sampled. Source data are provided as a Source Data file.

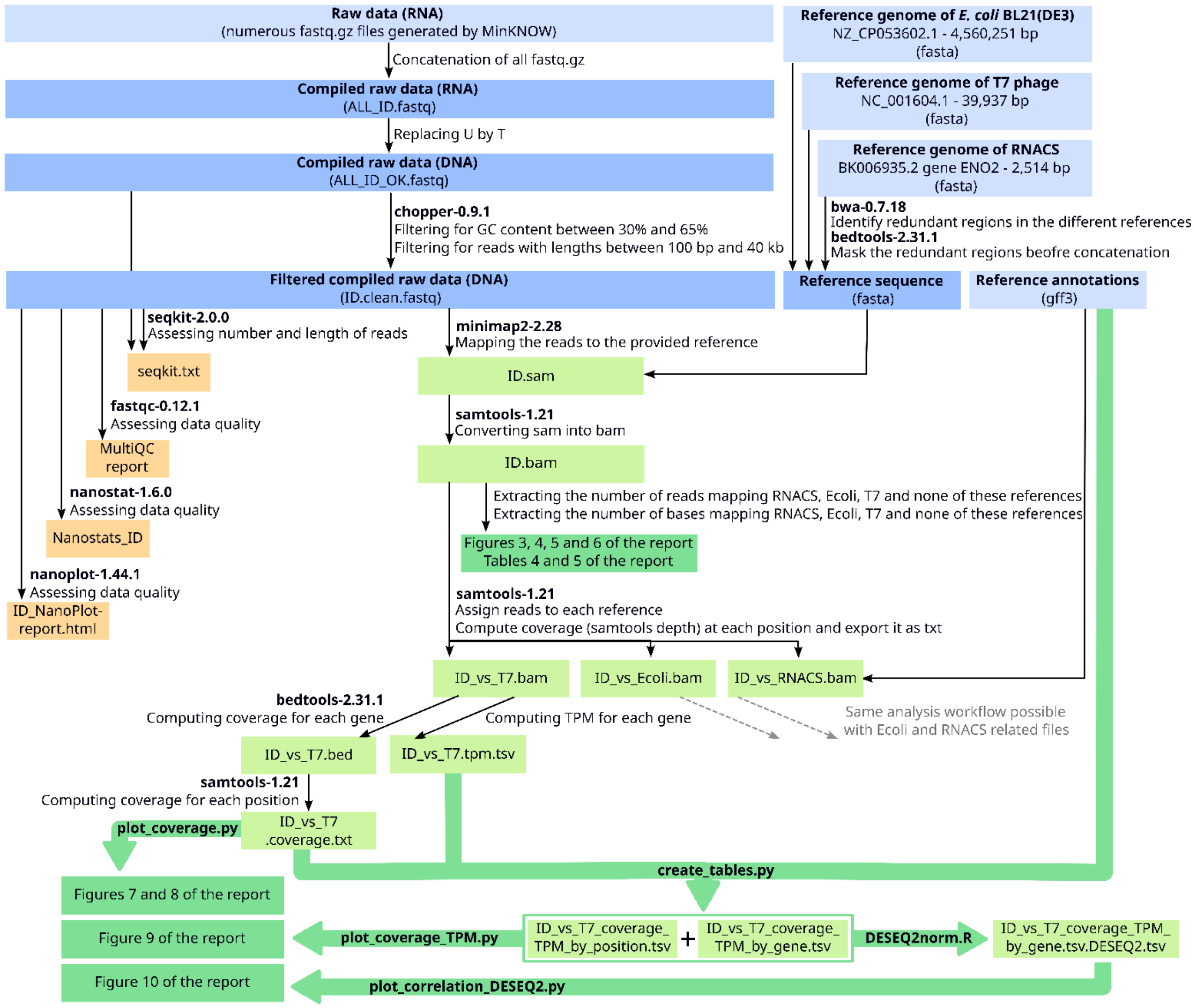

Supplementary Fig. 6 | RNA-seq data analysis workflow. ID refers to the flow cell identification number (e.g., FAZ55080), and REF indicates the reference genome used for the analysis (either "T7", "Ecoli", or "RNACS"). The scripts used for each step are described in the accompanying analysis report available on Recherche Data Gouv (https://doi.org/10.57745/BHTM60).

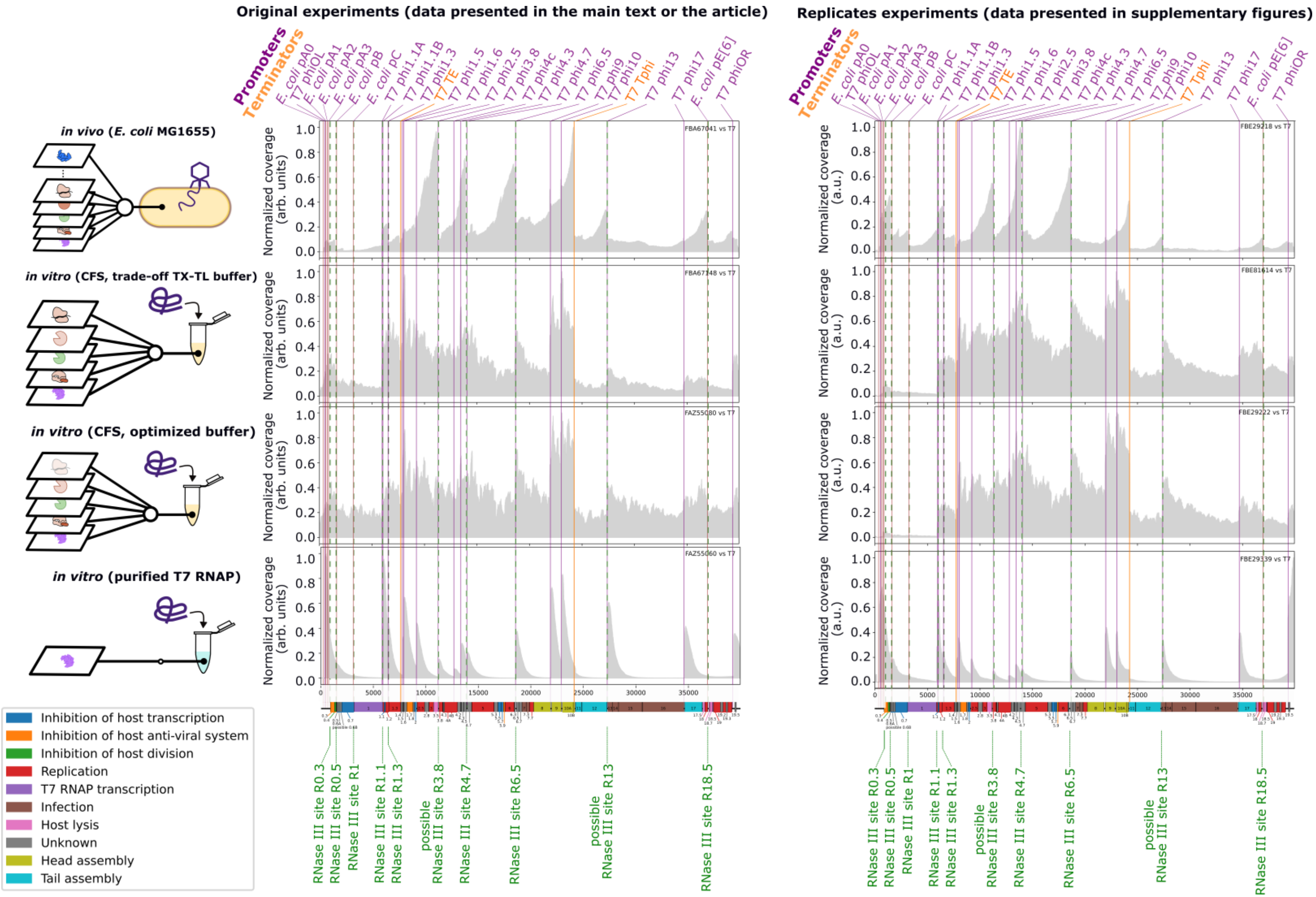

Supplementary Fig. 7 | Comparison of genome-wide expression profiles of T7 genome between replicates in each condition. On the left, schematic of the T7 genome expression *in vivo* and cell-free using the trade-off and mRNA-optimized buffers, as well as with purified T7 RNA polymerase. On the right, sequencing depth (i.e., the number of times each base in the genome has been sequenced), normalized by the maximum value within each sample, is indicated by grey shaded areas. Promoters and terminators position are shown in purple and orange respectively. The position of the genes on the chromosome are represented in grey under the plots. Source data are provided as a Source Data file.

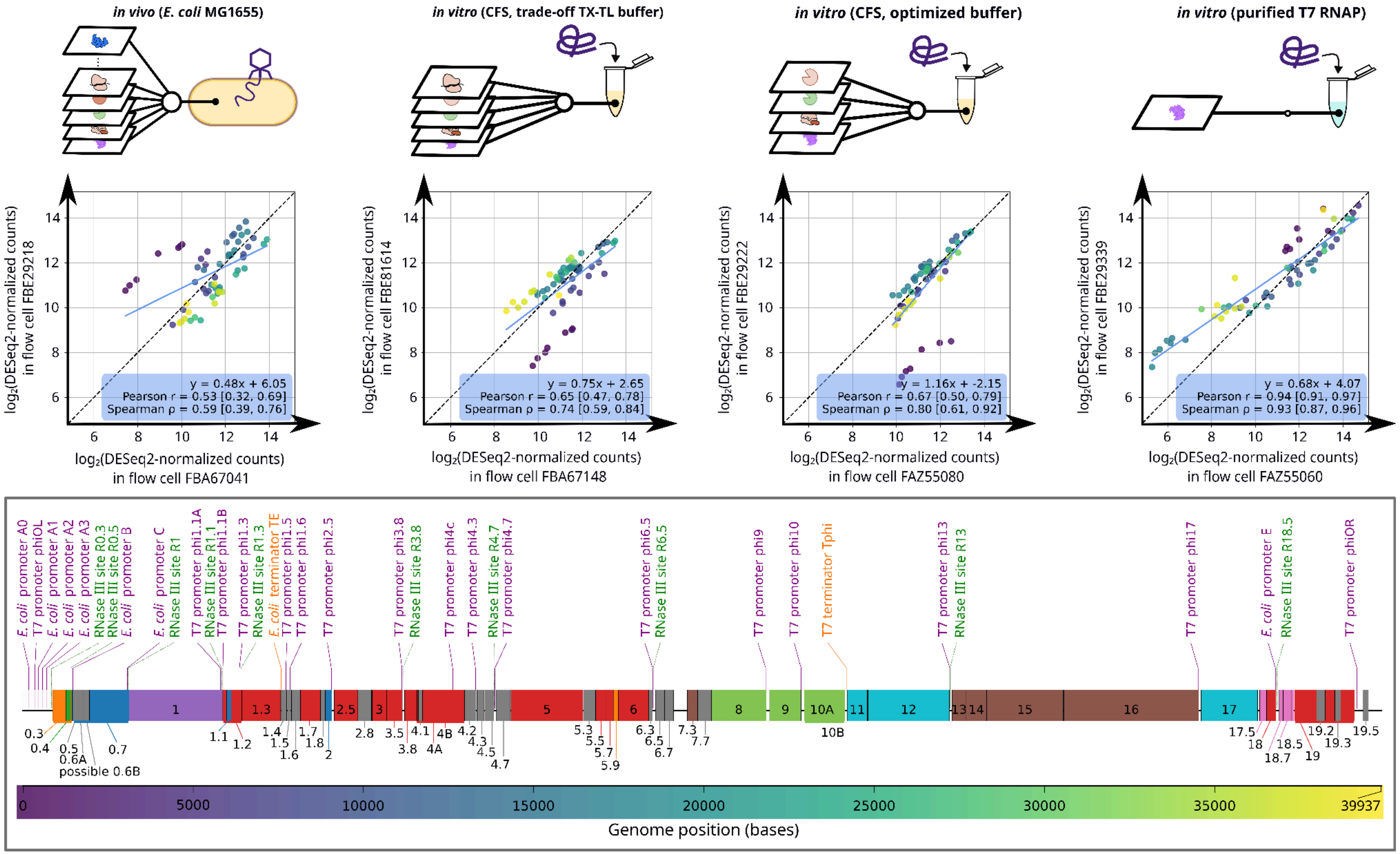

Supplementary Fig. 8 | Correlation of log2 DESeq2-normalized gene counts between replicates for each condition. Scatter plots show pairwise comparisons of log2-transformed DESeq2-normalized gene counts between *in vivo* infection and the trade-off buffer (left), the mRNA-optimized buffer (middle), and purified T7 RNAP (right). Each point represents one of the 60 T7 genes and is colored depending on its position on the T7 genome, as described on the legend below. The blue line indicates the linear regression fit (y = ax + b), and the dashed black line represents the identity line (y = x). Pearson's correlation coefficient (r) and Spearman's rank correlation coefficient (ρ) are reported in each panel. The 95% confidence intervals (CI) are shown in brackets; Pearson intervals were computed using a Fisher z-transformation, and Spearman intervals by bootstrap resampling (n = 1,000). Source data are provided as a Source Data file..

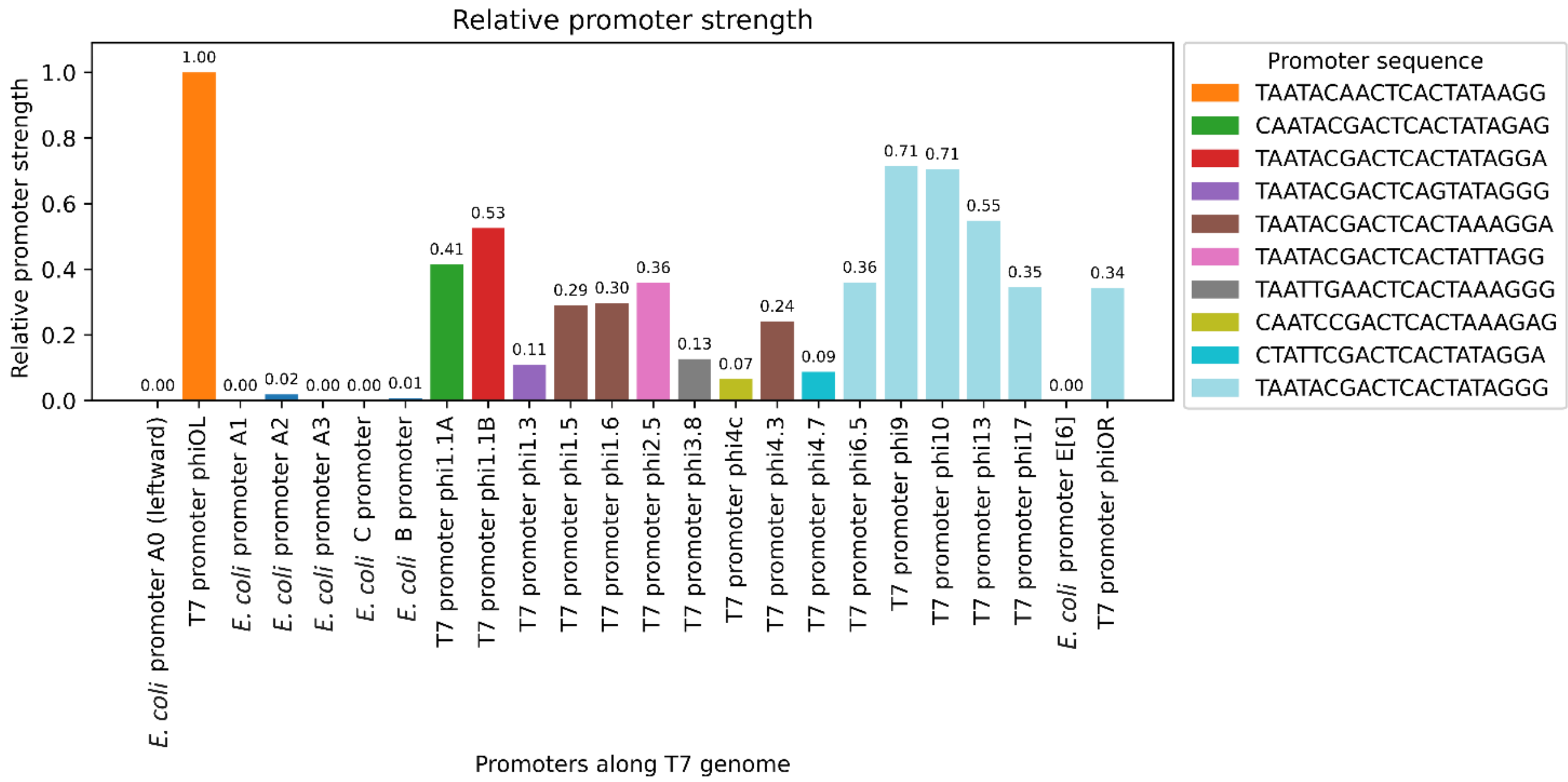

Supplementary Fig. 9 | Comparative analysis of T7 RNAP-specific promoter strengths in their native genomic context. Bar plot showing normalized promoter strengths derived from genome-wide RNA-seq coverage in the minimalist system. Promoter strength was quantified as the difference between the mean coverage from position +12 to +42 downstream of the transcription start site (TSS) of the annotated promoter and the mean coverage within the first 10 bases (position 0 to +10 relative to the TSS). This windowing strategy was chosen to (i) capture productive elongation downstream of promoter escape while avoiding the immediate promoter-proximal region, which is influenced by transcription initiation dynamics and polymerase pausing, and (ii) provide a local baseline for each promoter, thereby reducing the impact of upstream transcriptional read-through. Negative values were set to zero; this occurred only for the *E. coli* promoters A1, A3, C and E. Strength values were normalized to the maximum observed signal. Bars are colored according to promoter sequence, highlighting the relationship between sequence variation and transcriptional output within the native genomic context. Promoter strengths closely matched previous rankings based on [α-32P]ATP-labeled run-off transcripts[1] (**Supplementary Table 1**), and expanded the analysis to the 18 additional promoters not previously characterized. Source data are provided as a Source Data file.

Supplementary Table 1 | Comparison of the strength of 6 promoters previously measured in literature with the results of this study.

Promoter	relative_promoter_strength_LIT	ranking_LIT	relative_promoter_strength_EXP	ranking_EXP
T7 promoter phi10	1.00	1	1.00	1
T7 promoter phi13	0.79	2	0.78	2
T7 promoter phi1.1B	0.34	4	0.75	3
T7 promoter phi6.5	0.61	3	0.51	4
T7 promoter phi3.8	0.07	5	0.18	5
T7 promoter phi1.3	0.05	6	0.16	6

Column definitions: relative_promoter_strength_LIT and ranking_LIT refer to literature values and ranks using the same reference[1]; relative_promoter_strength_EXP and ranking_EXP refer to experimental

measurements and their corresponding ranks (1 = strongest, 6 = weakest). Relative promoter strength refers to promoter strength normalized by phi10.

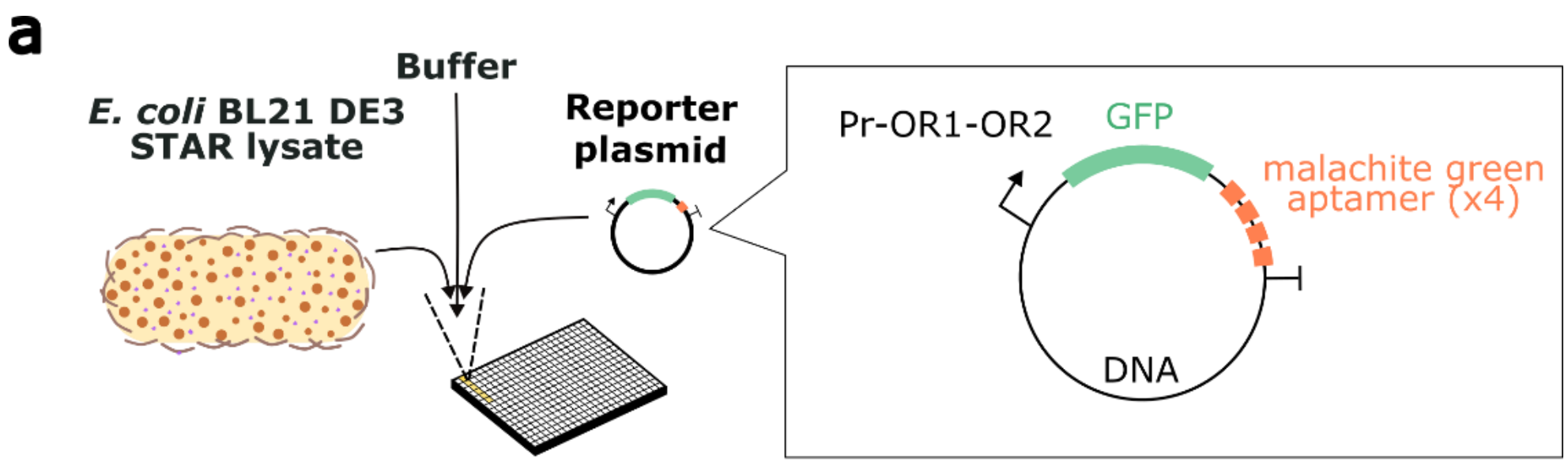

b

Compounds \ Buffer	Reference	Trade-off	Optimized
Mg-glutamate (mM)	4	9	27
K-glutamate (mM)	120	66	0
Amino acid (mM)	1.5	1.5	0
Spermidine (mM)	1	0.8	1
3-PGA (mM)	30	0	30
NTPs (mM)	1.5	8.3	14.4
PEG-8000 (%)	2	0	0
DNA (nM)	10	10	10
HEPES (mM)	50	50	50

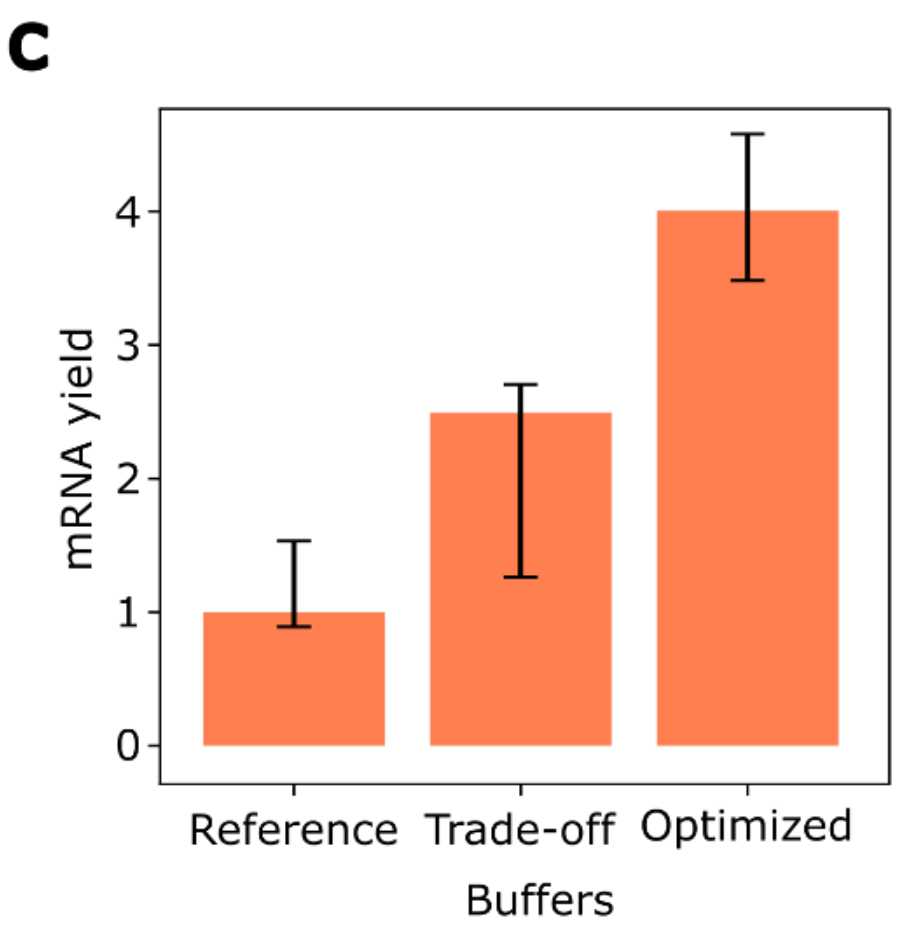

Supplementary Fig. 10 | Generalization of the optimization on *E. coli* RNAP. a, Schematic of the cell-free system setup: *E. coli* BL21 DE3 star lysate is complemented with buffer and a reporter plasmid. Reporter plasmid encodes *degfp* and four copies of the malachite green aptamer under the control of the Pr-ORI1-ORI2 for quantifying mRNA abundance via fluorescence. All measurements were performed in 384 well-plates with 10.5µL per well. **b**, Composition of the reference, trade-off and mRNA-optimized buffers. Compounds highlighted in yellow and in blue have increased and decreased concentrations compared to the reference, respectively. HEPES concentration was fixed during optimization. **c**, Coral bar plots show the median mRNA yield for each buffer composition. Error bars represent the 95% confidence interval across replicates (n=3 for plates 0-3; n=6 for plates 4-10). Despite the presence of four malachite green aptamer copies to enhance signal, overall fluorescence is lower using *E. coli* RNAP than T7 RNAP. Importantly, the relative order of buffer performance is preserved: the mRNA-optimized buffer, designed for T7 RNAP, increases mRNA yield by approximately 4-fold for *E. coli* RNAP, suggesting that the optimization may be beneficial beyond T7. Further testing would be required to confirm its general applicability. Source data are provided as a Source Data file.

Supplementary Table 2 | List of chemicals used in this study.

Product	Complete name	N° CAS	Chemical formula	M (g/mol)	Concentration of stock	Batch volume (mL)	Supplier	Reference	Storage
**Mg-glutamate**	L-Glutamic acid hemimagnesium salt tetrahydrate	18543-68-5	$C_{10}H_{16}MgN_2O_8 \cdot 4H_2O$	388.61	700 mM	5.00	Sigma-Aldrich	49605.000	RT
**K-glutamate**	L-Glutamic acid potassium salt monohydrate	6382-01-0	$C_5H_8KNO_4 \cdot H_2O$	203.23	3000 mM	30.00	Sigma-Aldrich	g1501	RT
**Spermidine**	Spermidine	124-20-9	$NH_2(CH_2)_3NH(CH_2)_4 NH_2$	145.25	100 mM	6.00	Sigma-Aldrich	s2626	-20°C
**3-PGA**	D-(-)-3-Phosphoglyceric acid disodium salt	80731-10-8	$C_3H_5Na_2O_7P$	230.02	1400 mM	3.92	Sigma-Aldrich	p8877	-20°C
**CTP**	Cytidine 5'-triphosphate disodium salt	36051-68-0	$C_9H_{14}N_3Na_2O_{14}P_3$	527.12	94 mM	1.75	Sigma-Aldrich	c1506	-20°C
**UTP**	Uridine 5'-triphosphate trisodium salt hydrate	19817-92-6	$C_9H_{12}N_2Na_3O_{15}P_3 \cdot xH_2O$	550.09	94 mM	1.75	Sigma-Aldrich	u6750	-20°C
**GTP**	Guanosine 5'-triphosphate sodium salt hydrate	36051-31-7	$C_{10}H_{16}N_5O_{14}P_3 \cdot xNa^+$	523.18	156 mM	1.75	Sigma-Aldrich	g8877	-20°C
**ATP**	Adenosine-5'-triphosphate·Na2 -salt	987-65-5	$C_{10}H_{14}N_5 O_{13}P_3 \cdot Na_2$	551.10	156 mM	1.75	Serva	10883.010	-20°C
**HEPES**	HEPES sodium salt	7365-45-9	$C_8H_{18}N_2O_4S$	238.30	2000 mM	50.00	Sigma-Aldrich	H3375	RT
**Malachite green**	Malachite green (oxalate)	2437-29-8	$C_{23}H_{25}N_2$ 1/2 $C_2H_2 O_4$ $C_2 H O_4$	927.03	1 mM	5.00	Fischer	M/1245/46	RT
**PEG-8000 (%)**	Polyethylene glycol	25322-68-3	$H(OCH_2CH_2)_nOH$	8000.00	20 %	100.00	Sigma-Aldrich	95172.000	RT

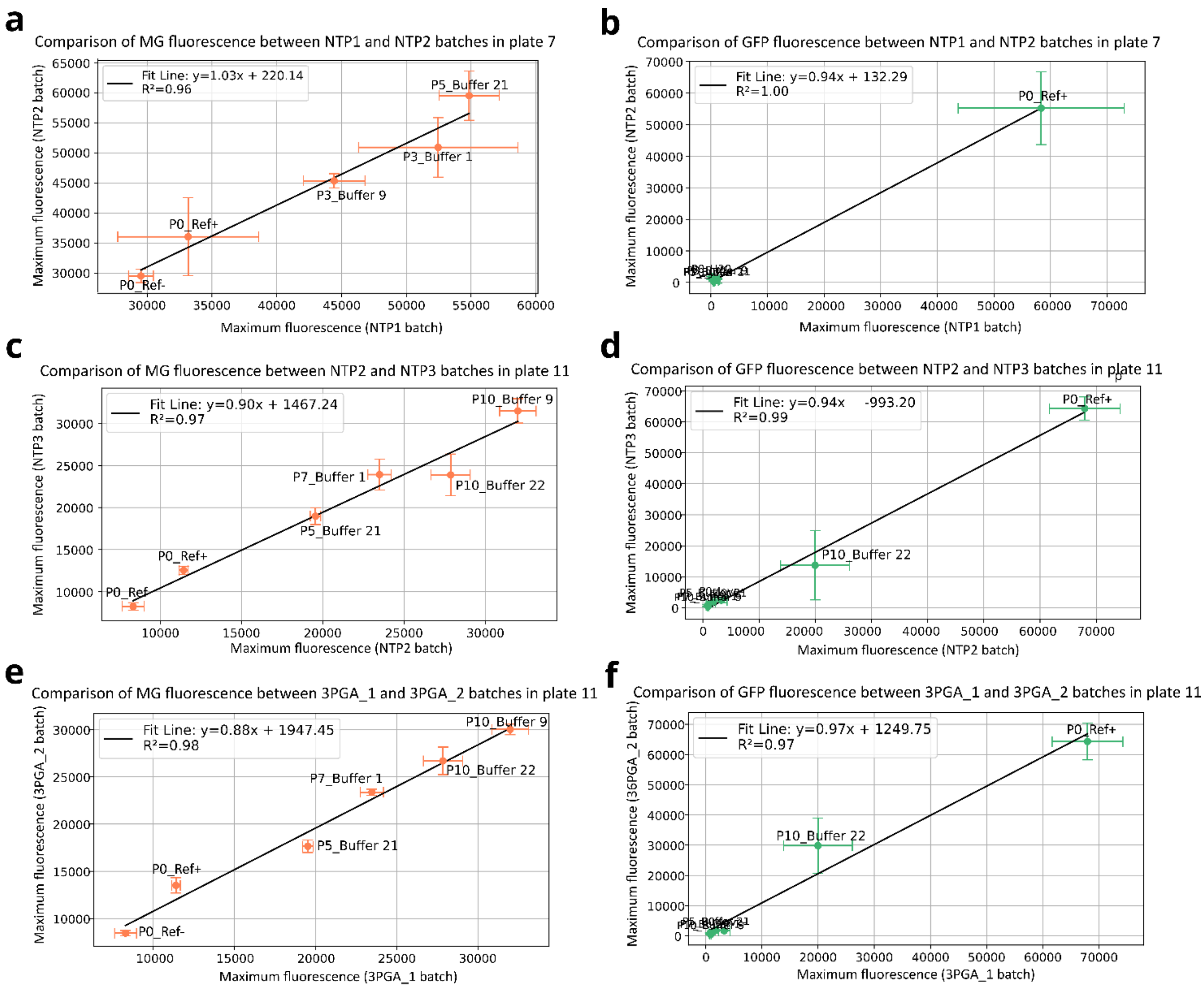

Supplementary Fig. 11 | Validation of new batches for compounds that had to be re-prepared during the experiment (NTPs and 3-PGA). Each plot compares mRNA or protein production in each buffer composition using the original batch (x-axis) versus a re-prepared batch (y-axis). A set of control buffers was tested in each plot, selected to span the full range of observed fluorescence values, whose composition are specified **Supplementary Data 1**.

a, **b**, Comparison of the impact of first and second batches of NTPs on mRNA (a) and protein (b) production.

c, **d**, Comparison of the second and third batches of NTPs for mRNA (c) and protein (d) production.

e, **f**, Comparison of the first and second batches of 3-PGA for mRNA (e) and protein (f) production.

Source data are provided as a Source Data file.

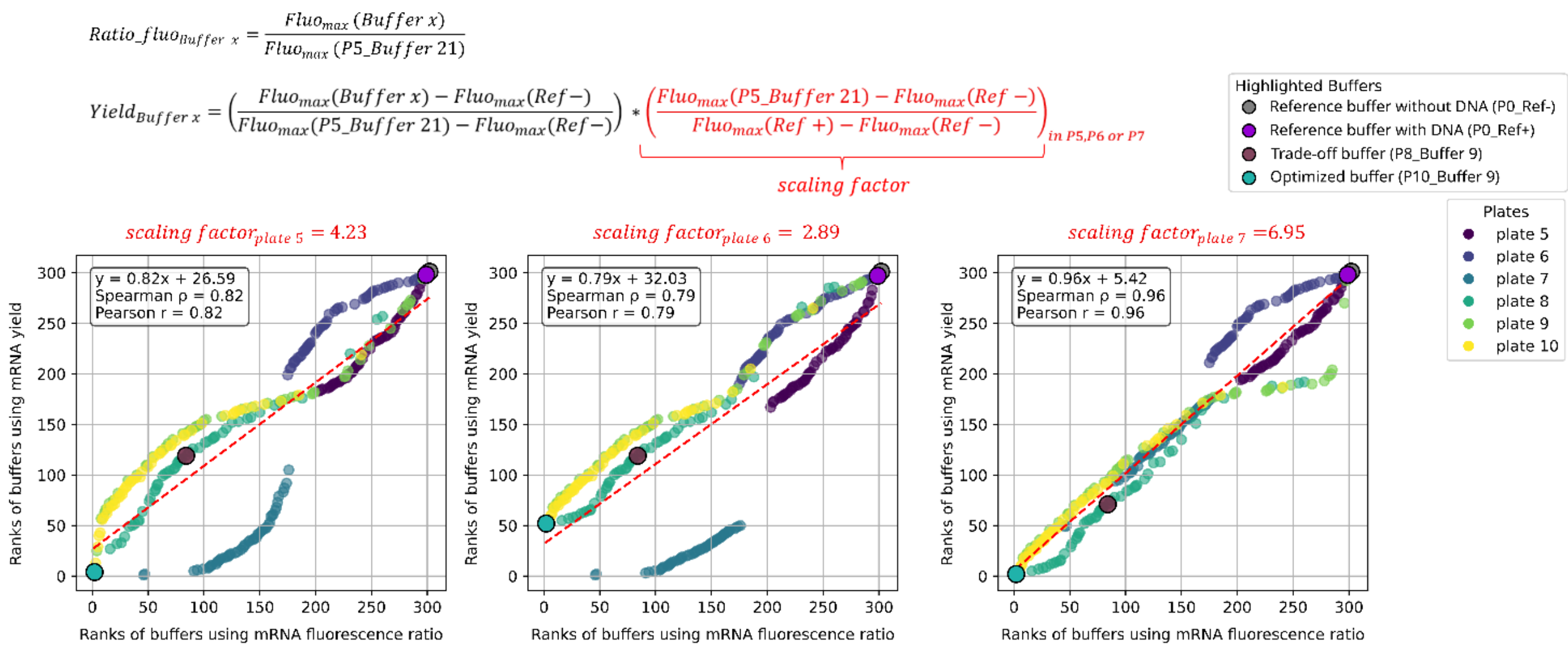

Supplementary Fig. 12 | Comparison of buffer rankings using scaling factor corrected mRNA yield across different correction plates. Scatter plots show the rank of each buffer (*MG_yield_rank*) computed from the scaling factor corrected yield versus the simplified fluorescence ratio (*ratio_fluo*). Each panel corresponds to a different *corr_plate* used to compute the scaling factor: plate 5 (left), plate 6 (middle), and plate 7 (right). Buffers highlighted in color represent specific references or controls (e.g., P0_Ref+, P0_Ref-, trade-off buffer, mRNA-optimized buffer), while other buffers are colored by plate. Trendlines indicate linear correlation, and Spearman (ρ) and Pearson (r) coefficients are displayed for each panel. The comparison shows that using P5_Buffer 21 in plate 7 as *corr_plate* yields the strongest agreement between rankings, supporting its use as the reference for scaling. Source data are provided with this paper.

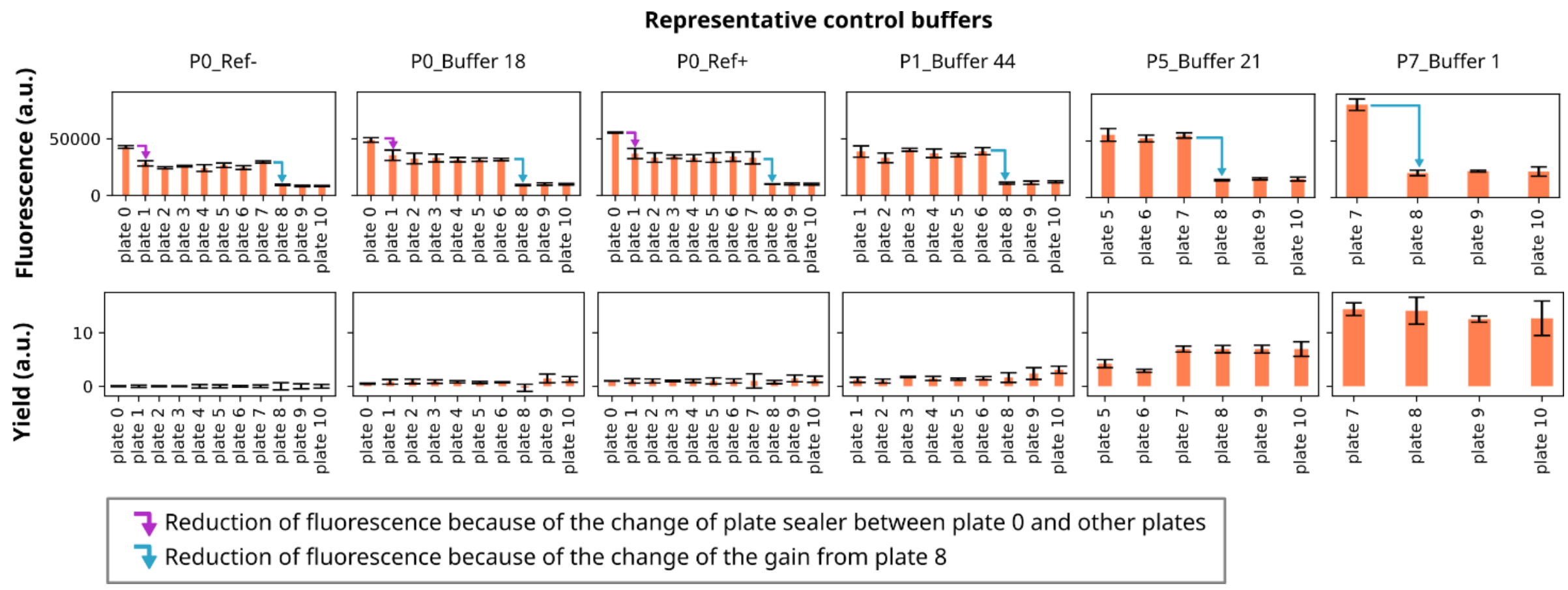

Supplementary Fig. 13 | Normalization of fluorescence measurement and experimental biases by the calculation of the yield. For a representative set of buffers covering the range from low to high fluorescence, the top row shows raw fluorescence values (arbitrary units), while the bottom row displays the corresponding yields (normalized fluorescence values; see **Methods**). Colored arrows highlight experimental biases that affect raw fluorescence but are corrected by normalization.
Source data are provided as a Source Data file.

a

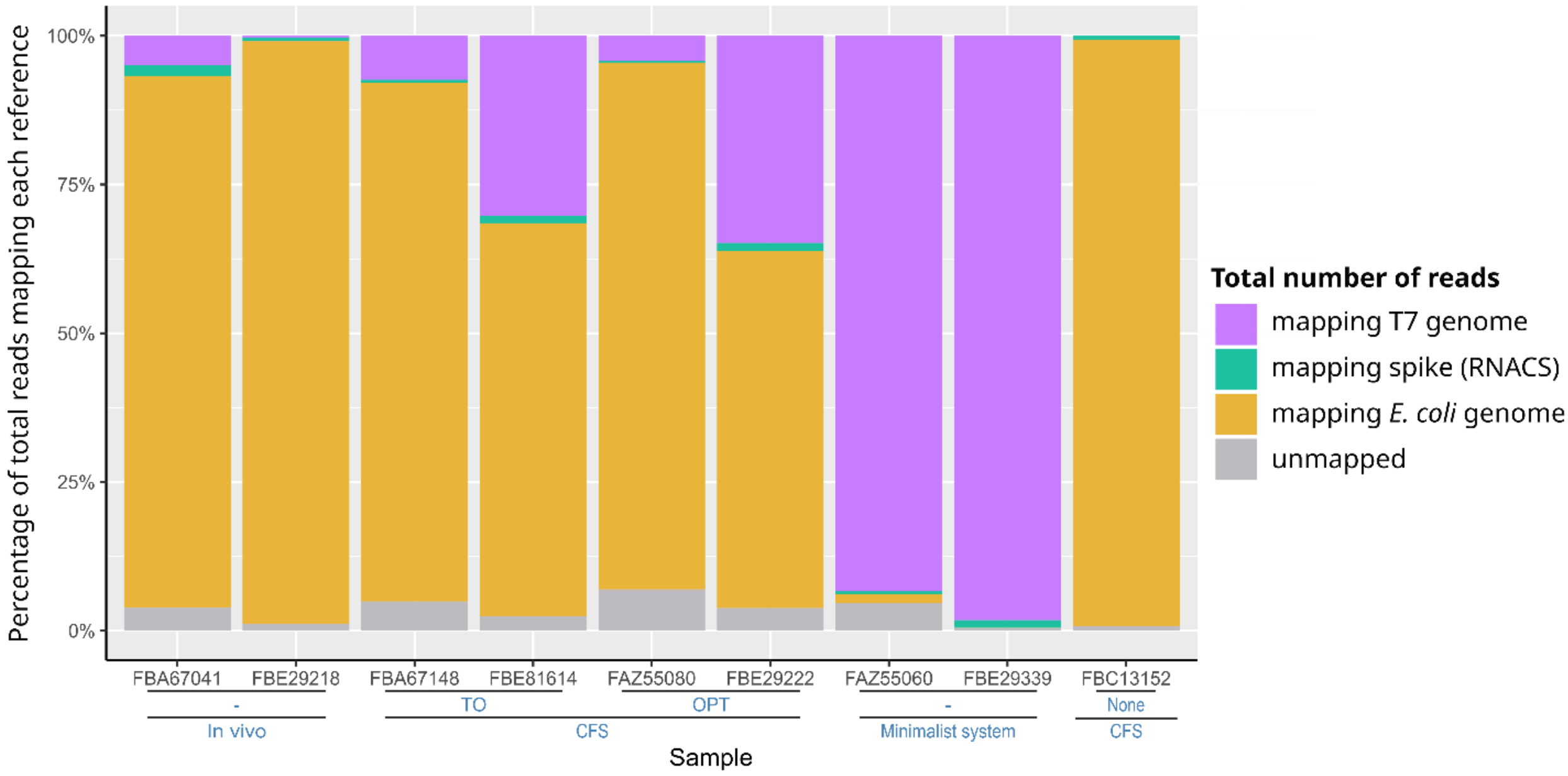

b

Sample	Description	total_reads	T7	RNACS	Ecoli	unmapped _reads
FBA67041	T7_in_vivo_10min	848274	41857	15280	758428	32709
FBE29218	T7_in_vivo_10min_2	3866180	13872	22148	3784609	45551
FBA67148	T7_CFS_tradeoff_P8bff9	6049445	449289	26147	5277291	296718
FBE81614	T7_CFS_tradeoff_P8bff9_2	2820952	855183	33786	1865170	66813
FAZ55080	T7_CFS_mRNAopt_P10bff9	13696690	568235	48098	12130344	950013
FBE29222	T7_CFS_mRNAopt_P10bff9_2	5983823	2083404	80942	3591822	227655
FAZ55060	T7_T7RNAP	11252272	10508901	51157	164372	527842
FBE29339	T7_T7RNAP_2	11897666	11687599	152842	1188	56037
FBC13152	Lysate (negative control without DNA)	4366226	11	27076	4306919	32220

Supplementary Fig. 14 | Number of reads per flow cell and proportion of reads mapping to the T7 genome. a, Relative or **b,** absolute number of reads. The flow cell ID is indicated for each sample as follows: FBA67041 and FBE29218, T7 genome expressed in vivo; FBA67148 and FBE81614, T7 genome expressed in CFS with the trade-off buffer; FAZ55080 and FBE29222, T7 genome expressed in a CFS with the mRNA-optimized buffer; FAZ55060 and FBE29339, T7 genome expressed in a minimalist CFS using purified T7 RNA polymerase; FBC13152, negative control containing only RNA present in the lysate. Reads mapping to the E. coli genome predominantly originate from ribosomal RNA present in the lysate.
Source data are provided as a Source Data file.

Supplementary references

1. Ikeda RA. The efficiency of promoter clearance distinguishes T7 class II and class III promoters. Journal of Biological Chemistry. 1992 Jun 5;267(16):11322–8. doi:10.1016/S0021-9258(19)49913-4
