# Supplementary material for: Active-learning-guided optimization of cell-free systems for genome-wide transcriptomic profiling reveals progressive layers of regulation
Source: Nat Commun. 2026 Jun 27;17:8065. doi: 10.1038/s41467-026-74559-y (PMC13454534; doi:10.1038/s41467-026-74559-y)
Supplement: Supplementary file 2 [file 41467_2026_74559_MOESM2_ESM.pdf]

Description of Additional Supplementary Files:

Supplementary Data 1: CSV file providing buffer compositions and corresponding fluorescence and yield measurements for MG and GFP across all active learning plates (0 to 10). Each row corresponds to a single buffer tested on a specific plate. The "Buffer" column provides the unique buffer identifier (e.g., "P3_Buffer 2"), while the "name_plate" column indicates the plate in which the buffer was tested (e.g., "plate 4"). The first eight columns report concentrations of buffer components: Mg-glutamate, K-glutamate, Amino acids, Spermidine, 3-PGA, NTPs, PEG-8000, and DNA (in mM, except PEG in % and DNA in nM). For both MG (malachite green aptamer) and GFP, columns *_fluo_1 to *_fluo_6 and *_yield_1 to *_yield_6 contain replicate fluorescence and yield values, respectively. Summary statistics are provided in the columns *_fluo_median, *_fluo_std, *_fluo_CI_low, and *_fluo_CI_high for fluorescence, and *_yield_median, *_yield_std, *_yield_CI_low, and *_yield_CI_high for yield. Columns well_1 to well_6 indicate the replicate well positions within each 384-well plate.
