# Supplementary material for: Active-learning-guided optimization of cell-free systems for genome-wide transcriptomic profiling reveals progressive layers of regulation
Source: Nat Commun. 2026 Jun 27;17:8065. doi: 10.1038/s41467-026-74559-y (PMC13454534; doi:10.1038/s41467-026-74559-y)
Supplement: Supplementary file 4 [file 41467_2026_74559_MOESM5_ESM.pdf]

nature portfolio

Peer Review File

Active-learning-guided optimization of cell-free systems for genome-wide transcriptomic profiling reveals progressive layers of regulation

Corresponding Author: Dr Olivier BORKOWSKI

This file contains all reviewer reports in order by version, followed by all author rebuttals in order by version.

Version 0:

Reviewer comments:

Reviewer #1

(Remarks to the Author)
The authors demonstrate selective optimization of a cell-free expression system to improve mRNA yields and illustrate the utility of this system for phage genome transcriptomics. The work builds upon numerous reports that different optimal conditions exist for transcription and translation in cell-free reaction environments. The novelty of this paper comes from using active learning-based optimization to explore the combinatorial reagent space and identify conditions in which high mRNA levels can be obtained for omics work. The utility of the developed formulation is then demonstrated by comparing T7 phage genome transcriptomics between the cell-free platform, a purified T7 RNA polymerase system, and in vivo phage infection. To my knowledge, this is the first demonstration of transcriptomics from a cell-free crude lysate system, complementing existing metabolomic and proteomic workflows. In addition, cell-free transcriptomics are shown to have a stronger correlation to the in vivo system, while the purified system shows no correlation at all.

The claims generally fit the provided data, the methodology appears sound, and enough detail is given to reproduce the work. However, increased discussion of why cell-free transcriptomics is important would be beneficial, particularly because genome-scale cell-free expression is relatively rare. Consideration of work from Vincent Noireaux, Vishwesh Kulkarni, and Eric van der Helm may be helpful here and needs to be better discussed. A few more comments:

Concerns
• There are some misleading statements, captions, and figures throughout the work. For example, the abstract claims that over 1,000,000 buffer conditions were explored, but the authors only tested 653 unique buffer formulations to explore a theoretical combinatorial space of over 1,000,000 conditions. This needs to be corrected, and directly clarified.
• Several figures (e.g., Fig. 1e) do not have axis units provided, and although these metrics are described fully in the methods and mentioned in the main body text, references or explanations should be included in the captions.
• Figure 2b appears to suggest that the E. coli ribosome is not active in the cell-free system, which is doesn’t make sense for a crude lysate platform. Is this true?
• The paper would benefit from grammatical revision in some places as well.
• The authors mention buffer concentration constraints due to viscosity of stock solutions and challenges with liquid transfer using the Echo liquid handler. Additional discussion about how these difficulties were mitigated would be informative and increase confidence in the accuracy and replicability of the results.
• An explanation of why the “optimized” formulation with low protein yield was chosen rather than the “trade-off” condition, which provided ~13x more mRNA without a decrease in protein yield, would be beneficial as well. This “trade-off” condition may provide interesting insights into genome transcription and translation simultaneously in the cell-free environment, which would be relevant to the study of phage behavior and more.
• I believe it is necessary to determine whether the optimized conditions also improve mRNA levels associated with endogenous RNA polymerases in the crude cell lysate. Can the authors please test and show this?

(Remarks on code availability)

Reviewer #2

(Remarks to the Author)

Summary

The manuscript presents a cell-free system (CSF) transcriptomics platform optimized via active learning (Bayesian optimization with Gaussian processes) over a combinatorial buffer space (>10^6 possibilities). By testing 653 conditions, the authors identify a buffer that boosts T7 RNAP–driven mRNA yield ~20-fold, facilitating RNA sequencing from CSF. The authors benchmark genome-wide T7 phage transcription across (1) a minimalist in vitro system (purified T7 RNAP), (2) E. coli BL21(DE3) lysate prepared using the optimized buffer, and (3) in vivo infection, while using coverage profiles to discuss RNA synthesis and degradation.
The introduced optimization links CSF with genome-wide transcriptomics while addressing a well-documented RNA yield bottleneck. The methodology is interesting, with potential generalization to other systems.

Major remarks

Abstract: While the abstract outlines the methodology and the observed increase in mRNA yield, it remains rather general. I recommend adding the following information: (1) state the key bottleneck in cell-free transcriptomics (insufficient RNA for sequencing), (2) specify the employed machine learning strategy, (3) state the optimized and trade-off buffer compositions, and (4) briefly note the insight gained from T7 phage expression analysis.

Quantitative validation of the mRNA yield increase: The mRNA yield metric relies on malachite green aptamer fluorescence maxima and a reference buffer. The relative yield metric is suitable for buffer optimization, but it is less ideal for quantitative claims of fold-change. The authors could strengthen the robustness of the reported 20-fold increase by providing absolute transcript concentrations (nM or molecules/well), for example via calibration with synthetic RNA standards, qPCR quantification of selected transcripts, or by spike-in probes.

Another methodological concern is the fluorescence normalization change during the experiment due to the adjustment of plate reader gain. This required replacing the reference buffer with an alternative buffer and incorporating a scaling factor. While this is a pragmatic solution, it is non-standard and may introduce bias. It is reassuring that the authors analyzed potential batch effects in Supplementary Figure 8. However, I recommend they also document the variability of the scaling factor across plates 5–7 to demonstrate its stability and reinforce confidence in the robustness of the reported yield increases.

Biological generality: Because the reporter plasmid is driven by a T7 promoter, the optimization process is likely tuned to T7 RNA polymerase activity. It is unclear whether the same buffer conditions would be optimal for transcription by other RNA polymerases. Furthermore, the study focuses almost entirely on T7 phage as a proof-of-concept. While the optimized buffer clearly enables robust transcription of the T7 genome, it remains uncertain how well these conditions generalize to other biological contexts. The authors state that their platform establishes a foundation for cell-free transcriptomics of complete bacterial genomes and even unculturable organisms, but no experimental evidence is provided beyond T7. It would significantly strengthen the manuscript to provide some validation outside of T7. If such experiments are not feasible within revision, a more cautious framing of the claims about generalizability would be appropriate.

Main text brevity: The main text is quite brief relative to the Supplementary Notes, and would benefit from incorporating more of that material directly. In particular, I suggest adding (1) a summary of the discussion presented in Supplementary Note 3 to contextualize the buffer elements, (2) the transcriptomic analysis of the trade-off buffer (Supplementary Figure 5), and (3) a summary of the promoter strength comparison presented in Supplementary Figure 6 and Supplementary Table 1.

Supplementary Note 1: The comparison across ML models is helpful, but it is unclear whether hyperparameters for MLP, RF, and XGBoost were tuned or left at defaults. Since these models are highly sensitive to parameter choice, a lack of tuning could bias the comparison in favor of GP. I recommend clarifying the hyperparameter optimization strategy and, if not performed, discussing this limitation explicitly.

Supplementary Note 2: This note provides a useful overview of how the optimization algorithm explored the combinatorial space. However, I encourage the authors to provide a more explicit description of how the exploration–exploitation balance in the acquisition function was tuned. This would strengthen the reproducibility of the workflow. Were adjustments made based on model uncertainty, yield plateauing, or visual inspection? Additionally, testing a subset of these underexplored regions would help validate that no higher-yield optima were inadvertently missed. A short discussion on unexplored regions should be added to the main text.

The text also hints at possible transcription–translation trade-offs. Since Figure 1f already indicates little-to-no correlation between mRNA and protein yields across the explored space, the authors should reference this point more explicitly.

Interpretation of degradation pathways: The manuscript attributes differences in coverage between CFS and in vivo to RNase III versus RNase E. This is plausible, but still correlative without direct validation (e.g., using RNase knockouts or inhibitors). At minimum, the limitations of inferring mechanism from coverage alone should be made clearer.

Statistical analyses: The main text reports medians with SD across replicates (n=3 for plates 0–3; n=6 for plates 4–10). I would also recommend presenting confidence intervals for key buffers (reference, optimized, and trade-off).

In Figure 1f, please report the Spearman correlation coefficient, along with the respective 95% CI. For other correlation panels (Figure 2d and Supplementary Figures 5c-e), report the number of evaluated genes, alongside 95% CIs for R.

RNA-seq analysis: The RNA-seq data analysis section is thorough and transparent, with tool versions, scripts, and intermediate files clearly documented. However, I see a few areas where the workflow could be strengthened.

Quality control currently relies on a Q>8 filter and GC/length filtering. It would strengthen the workflow to also report per-sample read length distributions and the proportion of reads retained after filtering. In addition, incorporating an adapter-trimming step would increase confidence in the downstream analyses.

While masking the T7 RNAP locus in E. coli avoids one obvious source of ambiguity, this approach may not capture other high-identity regions between T7 and E. coli. A more systematic strategy would provide stronger assurance that reads are being assigned correctly between references (e.g., repeat masking).

Please explicitly report the minimap2 parameters used for read aligning. When considering quantification, FPKM is known to

be biased, especially with long-read data. TPM or raw counts normalized with DESeq2/edgeR would be more appropriate for comparisons. Reads mapping to the ENO2 spike-in are mentioned but do not seem to be integrated into normalization. Please clarify the usage of the spike-in.
Finally, it appears that only one flow cell per condition was sequenced, without biological replicates. If true, this should be stated explicitly, and replicate-level mapping statistics or even minimal replication would be valuable to assess reproducibility of the transcriptomic profiles. If technical or biological replicates were included, it is unclear whether they were processed independently or pooled before mapping. Presenting replicate-level mapping statistics and TPM distributions would boost reproducibility.

Minor remarks
Title: Consider rephrasing to highlight the research scope and biological impact.
Lines 23-24: Consider changing to: "Lysate-based cell-free systems (CFS) provide a powerful platform for studying fundamental biological processes and molecular mechanisms".
Lines 28-30: Consider rephrasing to: "For example, previous experiments showed that production and metabolic burdens can be untangled by complementing cell-free systems and in vivo measurements".
Line 31: Remove "and parts, biosensing".
Line 33: Change "Genome expression" to "Genome-wide expression".
Supplementary Note 2, Line 54: Consider adding a sixth category: high NTP-s with low Mg-glutamate levels.
Supplementary Note 3, Line 83: Change "significative" to "significant".
Lines 64-65: The statement that PEG-8000, amino acids, and spermidine have no impact on mRNA abundance should be rephrased. Given the transcription-centric objective and the tested ranges, modest or interaction-mediated effects could be missed. Please rephrase to: "We did not observe a detectable effect of spermidine, amino acids, or PEG-8000 on mRNA abundance in CSF within the tested ranges". It would also be helpful if the authors could briefly discuss possible reasons for these findings (especially in the context of discordant results for spermidine in the published literature).
Supplementary Figure 3, Line 101, Supplementary Figure 4, Line 108, Supplementary Note 4, Line 110: Contain leftover text: "Error! Reference source not found".
Lines 65-66: Replace "the compositions which favored mRNA yields…" with: "most compositions which favored mRNA yields…".
Line 70: Change to "higher levels of Mg-glutamate and NTPs, but lower levels of K-glutamate, amino acids, and PEG-8000…".
Figure 1g: Include the trade-off buffer composition.
Line 94: Please clarify the term "T7 optimized buffer" (mRNA-optimized buffer?).
Supplementary Note 5: The definition of promoter strength based on coverage windows (+12–42 vs. 0–10) seems somewhat arbitrary, it would be useful to justify this choice. Additionally, the discussion on the genomic context remains descriptive, more explicit analysis of sequence motifs or neighboring structural features could add to the mechanistic insight.
Supplementary Note 5, Line 138: Change "Comparison" to Comparison of".
Lines 113-114: The authors attribute the 3′ coverage skew in vivo to the presence of RNase E, which is absent from CFS. While this is plausible, other E. coli ribonucleases also contribute to global mRNA turnover and could play a role in shaping transcript coverage. The authors might wish to acknowledge this.
Line 277: Please clarify the variable "*FluoJove+*" (*FluoRef+*?).
Line 303: Please explicitly provide the employed GP hyperparameters.
Lines 351-352: It is unclear whether the results obtained from E. coli RNAP were analysed as these results do not appear in the main text.
Supplementary Figure 10: I suggest presenting this information in a table rather in a barplot. The number overlay makes the plot cluttered.
Supplementary Figure 10, Line 181: Change "mapping" to "mapping to".
Supplementary Figure 10, Line 186: Change "originates" to "originate".
Line 440: Change "samtools" to "samtools depth".

(Remarks on code availability)

Reviewer #3

(Remarks to the Author)
I co-reviewed this manuscript with one of the reviewers who provided the listed reports. This is part of the Nature Communications initiative to facilitate training in peer review and to provide appropriate recognition for Early Career Researchers who co-review manuscripts.

(Remarks on code availability)

Version 1:

Reviewer comments:

Reviewer #1

(Remarks to the Author)

The authors have addressed my concerns. It's an exciting manuscript.

(Remarks on code availability)

Reviewer #2

(Remarks to the Author)
The authors have substantially strengthened the manuscript with additional experiments, improved statistical analyses, clearer methodological descriptions, and better overall readability. I have four minor comments remaining:
1) The description of promoter-related findings would benefit from further clarification. Specifically, the text in lines 285-290 is difficult to follow and should be rewritten for improved clarity and flow. In its current form, the connection between coverage profiles and promoter strength is not sufficiently explicit and several statements appear abrupt. It would also strengthen this section to incorporate a brief summary of the results presented in Supplementary Figure 9 and Supplementary Table 1. Finally, please correct the typo on line 287 ("pics" to "peaks").
2) The validation experiment using endogenous E. coli RNA polymerase is introduced for the first time in the Discussion (lines 361-368). Since this constitutes an important experimental result supporting the generalizability of the optimized buffers, it would be more appropriate to present these data in the Results section, with the corresponding Supplementary Material reference.
3) While the authors note that the improvement achieved with the optimized buffer relative to the reference buffer is smaller for E. coli RNAP than for T7 RNAP (~4-fold vs ~20-fold), this difference is substantial and could be discussed more explicitly. A similar point also applies to the trade-off buffer. These observations suggest that both buffer conditions may be at least partially specific to the T7 transcription system, and that the current evidence supporting broader generalizability remains limited.
4) References to the Supplementary Material are still broken in the main text, appearing as "Error! Reference source not found" instead of proper pointers. Please ensure this issue is resolved in the final version, as it significantly affects readability.

(Remarks on code availability)

Reviewer #3

(Remarks to the Author)
I co-reviewed this manuscript with one of the reviewers who provided the listed reports. This is part of the Nature Communications initiative to facilitate training in peer review and to provide appropriate recognition for Early Career Researchers who co-review manuscripts.

(Remarks on code availability)

Open Access This Peer Review File is licensed under a Creative Commons Attribution 4.0 International License, which permits use, sharing, adaptation, distribution and reproduction in any medium or format, as long as you give appropriate

credit to the original author(s) and the source, provide a link to the Creative Commons license, and indicate if changes were made.
In cases where reviewers are anonymous, credit should be given to 'Anonymous Referee' and the source.
The images or other third party material in this Peer Review File are included in the article's Creative Commons license, unless indicated otherwise in a credit line to the material. If material is not included in the article's Creative Commons license and your intended use is not permitted by statutory regulation or exceeds the permitted use, you will need to obtain permission directly from the copyright holder.
To view a copy of this license, visit https://creativecommons.org/licenses/by/4.0/

We thank the Reviewers for their valuable feedback and suggestions, which have greatly contributed to improving our manuscript. We have responded to each of their comments (**in bold**) in the text of the manuscript as described below, mirroring the original version that prompted the question (*in light grey*) and the corrected version where the passages written *in red* have been changed.

1 Reviewer #1

1.1 General remarks

The authors demonstrate selective optimization of a cell-free expression system to improve mRNA yields and illustrate the utility of this system for phage genome transcriptomics. The work builds upon numerous reports that different optimal conditions exist for transcription and translation in cell-free reaction environments. The novelty of this paper comes from using active learning-based optimization to explore the combinatorial reagent space and identify conditions in which high mRNA levels can be obtained for omics work. The utility of the developed formulation is then demonstrated by comparing T7 phage genome transcriptomics between the cell-free platform, a purified T7 RNA polymerase system, and in vivo phage infection. To my knowledge, this is the first demonstration of transcriptomics from a cell-free crude lysate system, complementing existing metabolomic and proteomic workflows. In addition, cell-free transcriptomics are shown to have a stronger correlation to the in vivo system, while the purified system shows no correlation at all.

The claims generally fit the provided data, the methodology appears sound, and enough detail is given to reproduce the work. However, increased discussion of why cell-free transcriptomics is important would be beneficial, particularly because genome-scale cell-free expression is relatively rare. Consideration of work from Vincent Noireaux, Vishwesh Kulkarni, and Eric van der Helm may be helpful here and needs to be better discussed.

We thank the reviewer for their thoughtful and constructive comments. We have expanded the Introduction to better highlight the importance of cell-free transcriptomics, particularly in the context of genome-scale expression studies with Vincent Noireaux, Vishwesh Kulkarni, and Eric van der Helm references: *"A few studies have used CFS to express entire genomes[13–15]. For example, Shin et al. used an E. coli lysate-based cell-free system to produce viable T7 phage virions from its genomic DNA, although transcriptomic analysis was not performed[13]. Taking a step further, Fujiwara et al. used CFS to express the genome of Thermus thermophilus and quantify the expression of a subset of genes using qPCR[16]."* (lines 61-63).

1.2 Major and minor remarks

• There are some misleading statements, captions, and figures throughout the work. For example, the abstract claims that over 1,000,000 buffer conditions were explored, but the authors only tested 653 unique buffer formulations to explore a theoretical combinatorial space of over 1,000,000 conditions. This needs to be corrected, and directly clarified.

We agree with the reviewer that the original wording in the abstract could be misleading. We carefully reviewed the manuscript to identify statements that might induce confusion and clarified them accordingly. In particular, we revised the abstract to explicitly distinguish between the theoretical combinatorial space and the experimentally tested conditions. The sentence *"To address this, we performed cell-free transcriptomics by developing a platform using an active learning workflow to explore over 1,000,000 buffer conditions."* was replaced with: *"We systematically explored a combinatorial space of over 1,679,616 possible buffer compositions and experimentally tested 653 compositions across ten iterative cycles."* (lines 21-23).

• Several figures (e.g., Fig. 1e) do not have axis units provided, and although these metrics are described fully in the methods and mentioned in the main body text, references or explanations should be included in the captions.

Fig. 1e in the previous version of the manuscript corresponds to Fig. 2b in the revised manuscript; the corresponding axis units have now been added on the left-hand side (line 175).

• Figure 2b appears to suggest that the *E. coli* ribosome is not active in the cell-free system, which is doesn't make sense for a crude lysate platform. Is this true?

Fig. 2b in the previous version of the manuscript corresponds to Fig. 5a in the revised manuscript. In this figure, the *E. coli* ribosome is intentionally depicted as inactive because translation does not occur under mRNA-optimized buffer conditions. This buffer lacks essential components for translation, including amino acids and K-glutamate, and our active learning experiments confirm the absence of protein production. The figure caption has been revised to clarify this point: *"In cell-free reactions using the mRNA-optimized buffer, translation is inactive because essential components, including amino acids and K-glutamate, are absent; the E. coli ribosome is shown in a transparent form to indicate lack of activity"* (lines 302–304).

• The paper would benefit from grammatical revision in some places as well.

We thank the reviewer for this suggestion. We carefully revised the manuscript to improve grammar and clarity throughout the text.

• The authors mention buffer concentration constraints due to viscosity of stock solutions and challenges with liquid transfer using the Echo liquid handler. Additional discussion about how these difficulties were mitigated would be informative and increase confidence in the accuracy and replicability of the results.

The manuscript has been revised to further detail how these technical constraints were addressed: *"The limits of the combinatorial space were constrained by technical requirements of the Echo machine. Indeed, each ingredient's stock concentration must be (i) high enough that the total volume of all ingredients remains below 10.5 µL, (ii) low enough to avoid exceeding solubility limits or causing excessive viscosity, and (iii) compatible with acoustic dispensing, such that each individual transfer falls within the 20-1500 nL range of the Echo liquid handler. Stock concentrations were selected to satisfy these criteria, and a drop test was performed to confirm that all ingredients could be reliably dispensed by the Echo 650."* (lines 107–113).

• An explanation of why the "optimized" formulation with low protein yield was chosen rather than the "trade-off" condition, which provided ~13x more mRNA without a decrease in protein yield, would be beneficial as well. This "trade-off" condition may provide interesting insights into genome transcription and translation simultaneously in the cell-free environment, which would be relevant to the study of phage behavior and more.

Results obtained with the "trade-off" buffer were previously presented in Supplementary Note 4. In the revised manuscript, this condition is now integrated into the main text to improve clarity and accessibility, including the Abstract, Results, and Discussion, as well as in Fig. 3, Fig. 5, and Fig. 6.

• I believe it is necessary to determine whether the optimized conditions also improve mRNA levels associated with endogenous RNA polymerases in the crude cell lysate. Can the authors please test and show this?

We agree that assessing mRNA levels associated with endogenous RNA polymerases is important. To address this, we performed an additional experiment using a new reporter plasmid; the results are presented in Supplementary Fig. 10. These findings have also been incorporated into the Discussion: sion: *"Because active learning was conducted exclusively with T7 RNAP, the resulting compositions*

might not be fully optimal for transcription driven by the endogenous E. coli RNAP, potentially restricting the applicability of the "mRNA-optimized" and "trade-off" buffers. Nevertheless, the active learning framework itself remains transferable. Transfer learning based on the T7 RNAP dataset could accelerate adaptation to endogenous polymerases, provided that the explored compositional space already captured conditions favorable to endogenous transcription. To test mRNA yield improvements based on the endogenous E. coli RNAP, we measured mRNA production from a plasmid carrying a strong constitutive E. coli promoter upstream of the gfp gene (Supplementary Fig. 10a). Both the optimized and trade-off buffers significantly increased mRNA yield compared to the reference buffer (4-fold 95% confidence interval of [3.48, 4.58] and 2.5-fold 95% confidence interval of [1.26, 2.7], respectively (Supplementary Fig. 10b), although the magnitude of improvement was lower than that observed with T7 RNA polymerase. Nevertheless, these results demonstrate that the identified buffer compositions also enhance mRNA levels produced with bacterial machinery, providing a for further species-specific refinement." (lines 354–367).

2 Reviewer #2 (Remarks to the Author)

2.1 General remarks

The manuscript presents a cell-free system (CSF) transcriptomics platform optimized via active learning (Bayesian optimization with Gaussian processes) over a combinatorial buffer space (>10^6 possibilities). By testing 653 conditions, the authors identify a buffer that boosts T7 RNAP–driven mRNA yield ~20-fold, facilitating RNA sequencing from CSF. The authors benchmark genome-wide T7 phage transcription across (1) a minimalist in vitro system (purified T7 RNAP), (2) *E. coli* BL21(DE3) lysate prepared using the optimized buffer, and (3) in vivo infection, while using coverage profiles to discuss RNA synthesis and degradation.

The introduced optimization links CSF with genome-wide transcriptomics while addressing a well-documented RNA yield bottleneck. The methodology is interesting, with potential generalization to other systems.

2.2 Major remarks

***Abstract:* While the abstract outlines the methodology and the observed increase in mRNA yield, it remains rather general. I recommend adding the following information: (1) state the key bottleneck in cell-free transcriptomics (insufficient RNA for sequencing), (2) specify the employed machine learning strategy, (3) state the optimized and trade-off buffer compositions, and (4) briefly note the insight gained from T7 phage expression analysis.**

To address this request, we substantially revised the abstract to make it more specific and informative, and to explicitly address each of the points raised:

1. First, we now clearly state the key bottleneck in cell-free transcriptomics. The revised abstract explicitly identifies insufficient mRNA abundance for RNA sequencing as the main limitation: *"their application to transcriptomics has been constrained by mRNA abundance levels that are too low for RNA sequencing."* (lines 18-19).
2. Second, we specify the machine learning strategy used in this work. While the original abstract referred only generically to an optimization platform, the revised version now explicitly describes the approach as *"To overcome this bottleneck, we developed an active learning-guided optimization workflow combining Bayesian optimization using gaussian processes with automated high-throughput experimentation."* (lines 19-21).
3. Third, we now report both the optimized and trade-off buffer compositions directly in the abstract. The revised text details the key buffer components and their relative levels in the optimized condition: *"This approach identified two buffer formulations suitable for RNA-seq:*

the "mRNA-optimized" and the "trade-off" buffers, both containing higher concentrations of Mg-glutamate and NTPs and lower concentration of K-glutamate and PEG-8000 than the reference buffer". (lines 23-26).

4. Finally, we expanded the abstract to briefly summarize the biological insight gained from the T7 phage expression analysis. The revised abstract now highlights how increasing system complexity restores regulatory layers, including RNase III-mediated mRNA maturation and distinct degradation patterns. It also emphasizes the key conclusion that promoter strength alone is insufficient to predict mRNA levels. *Using direct RNA sequencing, we then performed genome-wide transcriptomic profiling of the 40 kb T7 phage genome in CFS with either the mRNA-optimized or the trade-off buffers. The resulting transcriptomic profiles were compared with those obtained from a minimalist in vitro transcription system based on purified T7 RNA polymerase and from phage-infected bacteria. This comparative analysis revealed distinct regulatory layers. The minimalist system is a direct proxy for quantifying promoter strength in its genomic context but lacks RNA degradation processes observed in vivo, whereas CFS provide an accurate estimation of in vivo gene expression levels and reveal the position of mRNA maturation sites."* (lines 29-36).

***Quantitative validation of the mRNA yield increase:* The mRNA yield metric relies on malachite green aptamer fluorescence maxima and a reference buffer. The relative yield metric is suitable for buffer optimization, but it is less ideal for quantitative claims of fold-change. The authors could strengthen the robustness of the reported 20-fold increase by providing absolute transcript concentrations (nM or molecules/well), for example via calibration with synthetic RNA standards, qPCR quantification of selected transcripts, or by spike-in probes.**

We thank the reviewer for this helpful suggestion. To address this point, we performed an additional experiment in which mRNA was purified from cell-free reactions, allowing us to quantify transcript levels. The resulting data have been added to the manuscript (Supplementary Fig. 2) and the results accordingly updated: *"In total, 653 buffer compositions were tested, leading to a ~20-fold (95% confidence interval of [16.45, 21.33]) increase in mRNA yield in a composition termed the "mRNA-optimized buffer" (Fig. 2b). A 7.5-fold increase in mRNA yield was observed using mRNA purification in the optimized buffer reaction, and a strong correlation between yield obtained by the malachite green and mRNA purification across multiple buffer compositions (Supplementary Fig. 2). As both methods carry inherent biases, the mRNA fold-improvement likely falls between these two values. Furthermore, mRNA purification strengthened our confidence in the rank ordering of buffer compositions relative to mRNA yield (Supplementary Fig. 2, Supplementary Fig. 3)."* (lines 167-174).

Another methodological concern is the fluorescence normalization change during the experiment due to the adjustment of plate reader gain. This required replacing the reference buffer with an alternative buffer and incorporating a scaling factor. While this is a pragmatic solution, it is non-standard and may introduce bias. It is reassuring that the authors analyzed potential batch effects in Supplementary Figure 8. However, I recommend they also document the variability of the scaling factor across plates 5–7 to demonstrate its stability and reinforce confidence in the robustness of the reported yield increases.

We agree that this normalization step following the change in plate reader gain is delicate and warrants detailed clarification.

At the start of the active learning process (plate 0), malachite green fluorescence was close to background levels, requiring the plate reader gain to be set to its maximum sensitivity. As optimization progressed, fluorescence signals increased substantially, and by plate 7 several conditions exceeded the detection limit (100,000 a.u.), resulting in saturation. To continue the optimization beyond this point, reducing the gain was necessary to avoid signal overflow and enable accurate measurements.

However, after this adjustment, the reference buffer (P0_Ref+) used for yield calculation in plates 0–7 could no longer be distinguished from background. To address this, we used an alternative buffer (corr_buffer) with measurable fluorescence under both gain settings. Yields in plates 8–10 were calculated relative to this corr_buffer and rescaled using a scaling factor defined as the yield of corr_buffer relative to P0_Ref+ in a plate prior to the gain change (corr_plate).

We acknowledge that the scaling factor is not strictly constant. To evaluate its impact, we computed it using corr_buffer = P5_Buffer 21 and corr_plate = plates 5, 6, or 7 (Supplementary Fig. 12). Using an underestimated scaling factor would lead to an underestimation of the yields of buffers measured in plates 8–10, whereas an overestimated scaling factor would result in an overestimation of MG_yield values in these plates. To identify the most appropriate value for the scaling factor, we introduced a new metric $Ratio\_fluo_{Buffer\ x}$ not affected by the background fluorescence, which preserves the ranking of conditions within plates. This metric can only be calculated for plates 5–10, because P5_Buffer 21, used as corr_buffer, was first measured in plate 5 and is not available in plates 0–4. Supplementary Fig. 12 demonstrates that the scaling factor derived from plate 7 best preserves the ordering of buffer conditions defined by $Ratio\_fluo_{Buffer\ x}$. This choice is further supported by independent control buffers (P7_Buffer 1 and P7_Buffer 25), not used for normalization, whose corrected yields in plates 8–10 are consistent with their values in plate 7.

Overall, although this correction introduces some variability, it represents the most reliable solution to ensure continuity of the active learning process across gain settings. These explanations have been detailed in the Methods (lines 486-514):

"Starting from plate 7, the active learning process successfully identified buffer compositions that substantially increased mRNA production, as reflected by higher malachite green fluorescence. This increase required adjusting the gain on the plate reader, which made the original reference buffer with DNA (P0_Ref+) indistinguishable from background noise. To correct for this, we used an alternative reference buffer (corr_buffer) that was previously measured in plates with the old gain, and recalculated yields as:

$$Yield_{Buffer\ x} = \left(\frac{Fluo_{max}(Buffer\ x) - Fluo_{max}(Ref-)}{Fluo_{max}(corr\_buffer) - Fluo_{max}(Ref-)}\right) * scaling\ factor$$

Where the $scaling\ factor = \frac{Fluo_{max}(corr\_buffer, corr\_plate) - Fluo_{max}(Ref-, corr\_plate)}{Fluo_{max}(Ref+, corr\_plate) - Fluo_{max}(Ref-, corr\_plate)}$

This correction can be computed using a scaling factor derived from:

any buffer that was measured both in plate 8 (after the gain change) and in at least one plate before the gain change (e.g., corr_buffer = P5_Buffer 21),

and any plate prior to the gain change in which this buffer was measured (defined as corr_plate; e.g., P5_Buffer 21 in plate 5, 6, or 7).

To select the optimal correction, we chose P5_Buffer 21 as the corr_buffer because, among the control buffers retested in multiple plates, it was the first to remain clearly distinguishable from background at both gain settings. For the corr_plate, we compared the buffer rankings computed using (i) the scaling factor applied to the yield, and (ii) a simplified fluorescence ratio metric (ratio_fluo) that does not subtract the autofluorescence to allow consistent comparison across plates 5, 6, 7, 8, 9 and 10 at both gain settings:

$$Ratio\_fluo_{Buffer\ x} = \frac{Fluo_{max}(Buffer\ x)}{Fluo_{max}(P5\_Buffer\ 21)}$$

This comparison was performed for all buffers in plates 5-10. The best correlation between the yield-based ranks and ratio_fluo-based ranks was observed when using P5_Buffer 21 in plate 7 as the reference (Supplementary Fig 12). Using this correction, the final yield calculation for plates 8, 9 and 10 was:

$$Yield_{Buffer\ x} = \left(\frac{Fluo_{max}(Buffer\ x) - Fluo_{max}(Ref-)}{Fluo_{max}(P5\_Buffer\ 21) - Fluo_{max}(Ref-)}\right) * 6.95$$

where 6.95 is the scaling factor determined from P5_Buffer 21 in plate 7."

***Biological generality:* Because the reporter plasmid is driven by a T7 promoter, the optimization process is likely tuned to T7 RNA polymerase activity. It is unclear whether the same buffer conditions would be optimal for transcription by other RNA polymerases. Furthermore, the study focuses almost entirely on T7 phage as a proof-of-concept. While the optimized buffer clearly enables robust transcription of the T7 genome, it remains uncertain how well these conditions generalize to other biological contexts. The authors state that their platform establishes a foundation for cell-free transcriptomics of complete bacterial genomes and even unculturable organisms, but no experimental evidence is provided beyond T7. It would significantly strengthen the manuscript to provide some validation outside of T7. If such experiments are not feasible within revision, a more cautious framing of the claims about generalizability would be appropriate.**

We agree that assessing generality beyond T7 RNAP is important. To address this, we performed an additional experiment using a reporter plasmid transcribed by endogenous RNA polymerases. The results are presented in Supplementary Fig. 10 and have been incorporated into the Discussion: *"Because active learning was conducted exclusively with T7 RNAP, the resulting compositions might not be fully optimal for transcription driven by the endogenous E. coli RNAP, potentially restricting the applicability of the "mRNA-optimized" and "trade-off" buffers. Nevertheless, the active learning framework itself remains transferable. Transfer learning based on the T7 RNAP dataset could accelerate adaptation to endogenous polymerases, provided that the explored compositional space already captured conditions favorable to endogenous transcription. To test mRNA yield improvements based on the endogenous E. coli RNAP, we measured mRNA production from a plasmid carrying a strong constitutive E. coli promoter upstream of the gfp gene (Supplementary Fig. 10a). Both the optimized and trade-off buffers significantly increased mRNA yield compared to the reference buffer (4-fold 95% confidence interval of [3.48, 4.58] and 2.5-fold 95% confidence interval of [1.26, 2.7], respectively (Supplementary Fig. 10b), although the magnitude of improvement was lower than that observed with T7 RNA polymerase. Nevertheless, these results demonstrate that the identified buffer compositions also enhance mRNA levels produced with bacterial machinery, providing a for further species-specific refinement."* (lines 354–367).

***Main text brevity:* The main text is quite brief relative to the Supplementary Notes, and would benefit from incorporating more of that material directly. In particular, I suggest adding (1) a summary of the discussion presented in Supplementary Note 3 to contextualize the buffer elements, (2) the transcriptomic analysis of the trade-off buffer (Supplementary Figure 5), and (3) a summary of the promoter strength comparison presented in Supplementary Figure 6 and Supplementary Table 1.**

Owing to the change in target journal from Nature Methods to Nature Communications, substantial material was initially placed in the Supplementary Notes to comply with main-text length constraints. In the revised manuscript, we have incorporated the requested elements directly into the main text including:

1. A summary of Supplementary Note 3 is now included to contextualize the impact of individual buffer components on mRNA yield: *"The impact of buffer composition on mRNA yield. Testing 653 buffer combinations showed correlations between mRNA yield and the concentration of components, some of which confirmed by the scientific literature while others revealing novel observations (Supplementary Fig. 3). Our optimization consistently favored high concentrations of Mg-glutamate and NTPs, reflecting their synergistic roles as T7 RNA polymerase cofactor and substrate, respectively (Supplementary Fig. 4). This is consistent with the two-metal-ion model of transcription, in which two magnesium ions coordinate the nucleophilic attack of the 3'-OH on the α-phosphate of the incoming NTP, facilitating phosphodiester bond formation. As Mg2+ is chelated by nucleotides, the concentration of the two components must increase accordingly to avoid reducing T7 RNA polymerase efficiency[28,29]. A new finding is the optimal intermediate concentration of 3-PGA to maximize mRNA yield, the mechanism behind this observation will be investigated in future studies (Supplementary Fig. 4). In contrast, three compounds in the buffer, amino acids, spermidine, and PEG-8000, remained unconstrained by the active learning model suggesting that they have little influence on mRNA yield within the tested ranges (Supplementary Fig. 3). For amino acids this result was expected as they are not required for transcription. Although spermidine has been reported as a critical component for in vitro transcription[30], our data showed no benefit which may be explained by the presence of endogenous polyamines in the lysate that compensate for its absence. Additionally, since PEG-8000 is designed to enhance crowding in the cell-free reaction, the increased concentrations of other components may have mitigated its effects. Overall, our active-learning-guided optimization simultaneously validates established transcription conditions and showed unintuitive optimal buffer compositions."* (lines 185-205).

2. The transcriptomic analysis of the trade-off buffer, previously in Supplementary Figure 5, has been integrated to highlight how this condition balances high mRNA production with protein yield: *"In CFS, transcripts from T7 RNAP-dependent promoters were detected as well (Fig. 5a, middle rows). Uniform transcript coverage reflected balanced transcription and degradation rates. In T7 phage biology, mRNA maturation is driven by RNase III-mediated processing[35]. Unlike the minimalist system, CFS restores T7 mRNA maturation, which is likely mediated by endogenous RNase III (Fig. 5b, bottom and middle rows). Finally, read coverage from in vivo data was skewed toward the 3' end of transcripts, indicating an imbalance between mRNA production and degradation (Fig. 5a, top row). It suggests that mRNA production and/or degradation differs from that observed in CFS. One hypothesis for the difference in degradation is the absence of membrane-associated proteins. Among the ribonucleases of E. coli, RNase E is membrane-associated[36,37] and its absence in CFS1 could account for the observed differences."* (lines 289-298).

3. A summary of the promoter strength comparison, previously presented in Supplementary Figure 6 and Supplementary Table 1, has been added to provide a direct comparison of transcriptional activity across different promoter sequences: *"Interestingly, promoters whose sequences are TAATACGACTCACTATAGGG showed genome location-dependent expression, confirming the strong impact of the genomic context on promoter strength (Supplementary Fig. 9, Supplementary Table 1)."* (lines 286-289).

Supplementary Note 1: **The comparison across ML models is helpful, but it is unclear whether hyperparameters for MLP, RF, and XGBoost were tuned or left at defaults. Since these models are highly sensitive to parameter choice, a lack of tuning could bias the comparison in favor of GP. I**

recommend clarifying the hyperparameter optimization strategy and, if not performed, discussing this limitation explicitly.

We have clarified the hyperparameter optimization strategy in the revised manuscript (Methods): *"o compare these models, we applied repeated random sub-sampling validation, randomly splitting the dataset into 80% training and 20% testing subsets over 50 iterations, and evaluated performance using the coefficient of determination (R^2), which quantifies the proportion of variance in the observed data explained by the model. R^2 ranges from negative infinity to 1, where 1 indicates a perfect fit, 0 corresponds to predicting the mean, and negative values indicate worse-than-mean performance. All models (MLP, RF, XGBoost, and GP) were systematically tuned during the experiments. For each training subset, a grid search with 5-fold cross-validation was performed, and the best hyperparameters were then retrained on the full training set. The MLP was tuned for different hidden layer sizes (32, 64), (64), (32), and (10) to keep the network small and reduce overfitting. Random Forest was tuned for the number of estimators (10, 50) and maximum tree depth (5, 10), while XGBoost was tuned for the learning rate (0.01, 0.1), the number of boosting rounds (100, 500, 700), and maximum tree depth (3, 5, 7). Gaussian Processes were tuned for different kernel combinations (White, RBF, and Matérn) and for assumed noise variance (0.01, 0.05, 0.1). All models were trained and evaluated on identical data splits at each repetition to ensure a fair comparison. We observed that GP consistently outperformed the other algorithms, exhibiting the highest accuracy and reliability (Supplementary Fig. 1a)."* (lines 555-570).

Supplementary Note 2: **This note provides a useful overview of how the optimization algorithm explored the combinatorial space. However, I encourage the authors to provide a more explicit description of how the exploration–exploitation balance in the acquisition function was tuned. This would strengthen the reproducibility of the workflow. Were adjustments made based on model uncertainty, yield plateauing, or visual inspection?**

We have clarified our strategy for balancing exploration and exploitation in the revised manuscript: ***"****We next evaluated different acquisition strategies, including Upper Confidence Bound (UCB), expected improvement (EI), and probability of improvement (PI) (Supplementary Fig. 1b). During the initial iterations (plates 0–2), UCB was used with a manually adjusted exploration-exploitation balance to rapidly identify high-performing regions of the parameter space. To directly assess whether additional optima could be revealed through exploration, plate 3 was designed to compare two strategies in parallel: half of the tested buffer compositions were selected using a purely exploitative strategy (EI), while the other half were selected using a purely exploratory UCB strategy. Since exploratory sampling did not yield improvements beyond model predictions (Supplementary Fig. 1c), all subsequent iterations (plate 4) relied mainly on exploitative sampling using EI."* (lines 140-149).

We also completed the methods section**:** *"Optimization was driven by the Upper Confidence Bound (UCB) acquisition function, the expected improvement (EI) function, or the probability of improvement (PI) function, depending on the iteration. During the early cycles within a given combinatorial space, we used UCB with a manually adjusted exploration–exploitation balance to efficiently target high-potential regions of the search space. After two loops without noticeable improvement, we shifted the balance toward exploitation, using EI or PI, a choice guided by preliminary simulations showing that most genuine improvements occur within the first two to four loops (Supplementary Fig. 1b). Continuing exploration beyond this point tended to produce redundant evaluations, making exploitation the most practical strategy to accelerate convergence. Upon each redefinition of the combinatorial space, the acquisition function was reset to maintain balance, and subsequent iterations again favored exploitation when promising regions were rapidly identified. To ensure that high-yield optima were not missed, we compared two strategies on plate 3: half of the compositions were selected*

using a purely exploitative approach (EI), and half using a purely exploratory approach (UCB)." (lines 572-584).

Additionally, testing a subset of these underexplored regions would help validate that no higher-yield optima were inadvertently missed. A short discussion on unexplored regions should be added to the main text.

We have detailed our efforts to explore the combinatorial space, including:

1. initialization via Latin Hypercube Sampling: *"To initiate the active learning loop, the initial 100 experiments were selected using Latin Hypercube Sampling to ensure good coverage of the search space. LHS assumes the independence of input variables (X)."* (lines 526-528).
2. tuning of exploration-exploitation parameters: *"Optimization was driven by the Upper Confidence Bound (UCB) acquisition function, the expected improvement (EI) function, or the probability of improvement (PI) function, depending on the iteration. During the early cycles within a given combinatorial space, we used UCB with a manually adjusted exploration–exploitation balance to efficiently target high-potential regions of the search space. After two loops without noticeable improvement, we shifted the balance toward exploitation, using EI or PI, a choice guided by preliminary simulations showing that most genuine improvements occur within the first two to four loops (Supplementary Fig. 1b). Continuing exploration beyond this point tended to produce redundant evaluations, making exploitation the most practical strategy to accelerate convergence. Upon each redefinition of the combinatorial space, the acquisition function was reset to maintain balance, and subsequent iterations again favored exploitation when promising regions were rapidly identified. To ensure that high-yield optima were not missed, we compared two strategies on plate 3: half of the compositions were selected using a purely exploitative approach (EI), and half using a purely exploratory approach (UCB)."* (lines 572-584).

We note that identification of the global maximum cannot be guaranteed without testing the entire combinatorial space, which was not our objective. The goal was to use active learning to achieve, with a minimal number of experiments, sufficiently high mRNA yield for RNA-seq from cell-free reactions, which was successfully accomplished. We do not claim to have identified the global maximum.

The text also hints at possible transcription–translation trade-offs. Since Figure 1f already indicates little-to-no correlation between mRNA and protein yields across the explored space, the authors should reference this point more explicitly.

We have expanded the Results to explicitly address potential transcription-translation trade-offs: *"Most compositions which favored mRNA yields resulted in reduced protein yields, consistent with previous observations[31] (Fig. 3a, Supplementary Fig. 3). However, a "trade-off" buffer composition demonstrated that high mRNA abundance does not necessarily come with low protein production (Fig. 3a). Moreover, as the optimization algorithm concentrated on regions predicted to enhance mRNA yield large regions of the combinatorial space still remained underexplored. This partial coverage was also influenced by the intentional reduction of amino acid concentrations in plates 9 and 10, based on their minimal impact on mRNA output in earlier rounds. Several regions remain underexplored, including combinations of low DNA with elevated NTPs or Mg-glutamate, elevated amino acids with high NTPs, high K-glutamate with either elevated NTPs or Mg-glutamate and elevated NTPs with low Mg-glutamate (Supplementary Fig. 5). Notably, a high K-glutamate concentration has an important role in for ribosome structure and function[32]. Thus, although high protein yield can reduce mRNA yield, we cannot conclusively determine whether competition between translation and transcription limits protein production. If such competition exists, some compounds might appear beneficial not because*

they directly enhance transcription, but because they reduce translation, an energetically costly process[33].” (lines 207-222).

Interpretation of degradation pathways: **The manuscript attributes differences in coverage between CFS and *in vivo* to RNase III versus RNase E. This is plausible, but still correlative without direct validation (e.g., using RNase knockouts or inhibitors). At minimum, the limitations of inferring mechanism from coverage alone should be made clearer.**
We have clarified that the attribution of differences in coverage between CFS and in vivo to RNase III versus RNase E is correlative. The revised manuscript explicitly acknowledges the limitations of inferring specific degradation mechanisms from coverage data alone and avoids overinterpretation: *“It suggests that mRNA production and/or degradation differs from that observed in CFS. One hypothesis for the difference in degradation is the absence of membrane-associated proteins. Among the ribonucleases of E. coli, RNase E is membrane-associated[36,37] and its absence in CFS1 could account for the observed differences.”* (lines 295-298).

Statistical analyses: **The main text reports medians with SD across replicates (n=3 for plates 0–3; n=6 for plates 4–10). I would also recommend presenting confidence intervals for key buffers (reference, optimized, and trade-off).**
We thank the reviewer for this suggestion. Because fluorescence data are summarized using the median and sample sizes are small (n=3–6), parametric confidence intervals based on the mean ± 1.96 × SD / √n are not appropriate. We therefore computed non-parametric bootstrap confidence intervals on the median. For each buffer, well-level fluorescence values were resampled with replacement (1,000 iterations), the median was computed for each resample, and the 2.5th and 97.5th percentiles of the resulting distribution were used to define a 95% confidence interval. This approach does not rely on distributional assumptions and is well suited to the use of the median as an estimator.

In the code *From_biotek_data_to_active_learning_vf.py*, the following changes were made:

- the function **calc_median_std_per_buffer** was modified to return a DataFrame containing the median, standard deviation, and 95% bootstrap confidence interval for each buffer;
- the function **barplot_one_plate** was modified to allow the user to select whether error bars represent standard deviation or confidence intervals. The figure titles explicitly indicate which type of error bars is plotted;
- The function **calc_yield_NaN** was modified to return a DataFrame containing the median, standard deviation, and 95% bootstrap confidence interval for each buffer;
- The function **plot_barplot_heatmap_compil_plate_per_plate** was modified to allow the user to select whether error bars represent standard deviation or confidence intervals. The figure y label explicitly indicate which type of error bars is plotted;
- In the section **#%% [protein/MG] COMPILATION OF COMPLETE DATA FILES ACROSS PLATES**, the list **list_index** was updated to include columns corresponding to confidence intervals.

All figures in the manuscript now display error bars corresponding to confidence intervals rather than standard deviation. Supplementary Table 3 provides the median, standard deviation, and 95% confidence interval for each buffer. We also report confidence intervals for the key buffers (reference, optimized, and trade-off) in the text, in addition to medians.

In Figure 1f, please report the Spearman correlation coefficient, along with the respective 95% CI.
We thank the reviewer for this suggestion. We computed the Spearman correlation coefficient between mRNA and protein yields across plates, which quantifies monotonic relationships without assuming linearity. To estimate uncertainty, we used a non-parametric bootstrap approach: well-level data were resampled 10,000 times with replacement, the Spearman coefficient was recalculated for

each resample, and the 2.5th and 97.5th percentiles of the bootstrap distribution defined the 95% confidence interval. Missing values were excluded from the calculation.

In From_biotek_data_to_active_learning_vf.py, the function **plot_correlation_by_plate_labels** was updated to display the Spearman coefficient and its 95% CI directly on the plot (Fig 3a top-right corner), while retaining color coding by plate and highlighting key buffers. Figure 1f now reports both the correlation and its uncertainty.

For other correlation panels (Figure 2d and Supplementary Figures 5c-e), report the number of evaluated genes, alongside 95% CIs for R.
We have updated the correlation analyses and figure legends to report the number of evaluated genes and 95% confidence intervals for the correlation coefficients.

- **Fig. 6** in the revised manuscript (previously Fig. 2d) is now labeled as: *"Each point represents one of the 60 T7 genes. The blue line indicates the linear regression fit (y = ax + b), and the dashed black line represents the identity line (y = x). Pearson's correlation coefficient (r) and Spearman's rank correlation coefficient (ρ) are reported in each panel. The 95% confidence intervals (CI) are shown in brackets; Pearson intervals were computed using a Fisher z-transformation, and Spearman intervals by bootstrap resampling (n = 1,000)."* (lines 331-335).
- **Supplementary Fig. 8** in the revised manuscript (previously Supplementary Fig. 5c–e) is now labeled as: *"Each point represents one of the 60 T7 genes and is colored depending on its position on the T7 genome, as described in the legend below. The blue line indicates the linear regression fit (y = ax + b), and the dashed black line represents the identity line (y = x). Pearson's correlation coefficient (r) and Spearman's rank correlation coefficient (ρ) are reported in each panel. The 95% confidence intervals (CI) are shown in brackets; Pearson intervals were computed using a Fisher z-transformation, and Spearman intervals by bootstrap resampling (n = 1,000)."* (lines 56-60 of supplementary material).

RNA-seq analysis: **The RNA-seq data analysis section is thorough and transparent, with tool versions, scripts, and intermediate files clearly documented. However, I see a few areas where the workflow could be strengthened.**

- **Quality control currently relies on a Q>8 filter and GC/length filtering. It would strengthen the workflow to also report per-sample read length distributions and the proportion of reads retained after filtering.**

Per-sample read length distributions and the proportion of reads retained after filtering have now been added. Specifically, Table 2 ("Raw reads information") reports counts before filtering, and Table 3 ("Cleaned reads information") reports counts after filtering. This addition ensures full transparency of the quality control workflow.

- **In addition, incorporating an adapter-trimming step would increase confidence in the downstream analyses.**

We verified using Porechopper that adapters had already been removed by MinKNOW prior to data export, so no additional trimming was necessary within our workflow. This step is performed by default in the version 24.06.5 of MinKNOW, as indicated in the Methods section.

- **While masking the T7 RNAP locus in *E. coli* avoids one obvious source of ambiguity, this approach may not capture other high-identity regions between T7 and E. coli. A more systematic strategy would provide stronger assurance that reads are being assigned correctly between references (e.g., repeat masking).**

To ensure unbiased assignment of reads between references, we added a systematic step to the workflow in which BWA was used to align reference DNA sequences and identify any high-identity regions between coding sequences of different genomes. Applying this approach to the T7 and E. coli BL21 DE3 reference genomes confirmed that the only region of potential ambiguity was the T7 RNAP gene, which we had initially identified manually. This additional step provides stronger assurance that reads are correctly assigned between the two references.

- **Please explicitly report the minimap2 parameters used for read aligning.**

As indicated in Figure 1 of the HTML report deposited on the DataGouv repository and in the Methods, we used version 2.28 of Minimap2 for read alignment. The exact command and parameters are provided in the HTML report (https://doi.org/10.57745/BHTM60):

"# minimap2 index
conda activate minimap2-2.28
minimap2 3refs.fasta -x ava-ont -d 3refs.mmi
conda activate samtools-1.21 --stack
*for i in *clean.fastq.gz ; do id=$(echo $(basename $i | cut -d '.' -f 1)) ; minimap2 -ax map-ont -a 3refs.mmi $i --secondary=no -t 24 |samtools view -bS -F 2048 - |samtools sort - > ${id}.bam ; done*
conda deactivate
conda deactivate"

- **When considering quantification, FPKM is known to be biased, especially with long-read data. TPM or raw counts normalized with DESeq2/edgeR would be more appropriate for comparisons.**

We thank the reviewer for this suggestion. We have updated Fig. 6 and Supplementary Fig. 9 to use DESeq2-normalized counts instead of FPKM. The Methods have been revised to include the following explanation: *"TPM quantification. Gene expression levels were estimated from the BAM alignments using BEDTools29. Output included transcript-level TPM values stored in "ID_vs_REF.tpm.tsv", calculated as follows:*

$$TPM_i = \frac{FPKM_i}{\sum_j FPKM_j} * 10^6 \qquad with\ FPKM_i = \frac{counts_i * 10^9}{length_i * total\_counts}$$

DESeq2 quantification. To compare gene expression between samples, a custom R script (DESEQ2norm.R) was used to normalize counts with DESeq2. Values were stored in files named "ID_vs_REF_coverage_TPM_by_gene.tsv.DESEQ2.tsv".

*Data integration and visualization. Python scripts were used to integrate and visualize expression data. The script "create_tables.py" merged per-base coverage with TPM values, producing two output files: "ID_vs_REF_coverage_TPM_by_position.tsv" and "ID_vs_REF_coverage_TPM_by_gene.tsv", summarizing base-level and gene-level data, respectively. The script "plot_coverage.py" was used to generate coverage plots for each sample (**Error! Reference source not found.** and **Error! Reference source not found.**7). The script "plot_correlation_DESEQ2.py" was used to compare DESeq2-normalized expression values between flow cells (**Error! Reference source not found.** and **Error! Reference source not found.**8)."* (lines 725-738).

- **Reads mapping to the ENO2 spike-in are mentioned but do not seem to be integrated into normalization. Please clarify the usage of the spike-in.**

The ENO2 spike-in (RNA Control Strand, RNACS) was added by following the recommendations of the Oxford Nanopore Technologies direct RNA sequencing kit, with a constant nominal amount introduced into each library. During read classification, we therefore included a specific category for reads

mapping to the spike-in, alongside reads mapping to the T7 genome, the E. coli genome, or remaining unmapped. However, upon retrospective analysis, we observed that the RNACS was not stable over time. Specifically, measurements of RNACS-only tubes using Qubit revealed variability in RNA concentration depending on storage duration, which could introduce bias across transcriptomic experiments performed at different time points. For this reason, we did not use the spike-in for normalization. Importantly, normalization to the spike-in was not required for the analyses presented in this study, as our comparisons relied on relative expression patterns and coverage profiles within and across conditions rather than on absolute RNA quantification. We have clarified this point:*" Although RNACS was included as a spike-in following the Oxford Nanopore Technologies Direct RNA Sequencing Kit recommendations, retrospective analysis revealed that it was not stable over time: measurements of RNACS-only tubes using Qubit showed variable RNA concentrations depending on storage duration. For this reason, spike-in normalization was not applied. This did not affect the analyses presented here, as all comparisons were based on relative expression patterns and coverage profiles rather than absolute RNA quantities."* (lines 716-721).

- **Finally, it appears that only one flow cell per condition was sequenced, without biological replicates. If true, this should be stated explicitly, and replicate-level mapping statistics or even minimal replication would be valuable to assess reproducibility of the transcriptomic profiles. If technical or biological replicates were included, it is unclear whether they were processed independently or pooled before mapping. Presenting replicate-level mapping statistics and TPM distributions would boost reproducibility.**

We thank the reviewer for this important comment. In the original version of the manuscript, each RNA sequencing experiment was indeed performed only once. To strengthen our conclusions and improve confidence in the results, we have now duplicated all transcriptomic experiments. Each replicate was processed independently, from RNA production through RNA purification, library preparation, sequencing and mapping. The reproducibility between replicates is presented in the new Supplementary Fig. 8 & 9, which shows consistent mapping statistics and highly similar transcriptomic profiles across duplicates as described in the results *"Repeating our experiments revealed an interesting pattern in both CFS (optimized and trade-off) and in vivo conditions, the first 7 genes of the T7 genome showed high variability in expression between experiments. This shows that CFS captures the same variability as observed in vivo, which is not the case with the minimalist system (Supplementary Fig. 8). Eventually, our comparative analysis establishes cell-free systems as powerful platforms that bridge the gap between simplified reconstituted systems and complex in vivo environments, enabling systematic investigation of gene expression mechanisms."* (lines 320-326). Importantly, all key observations and conclusions of the study remain unchanged regardless of which replicate is considered. We also completed the methods: *"All figures presented in the main text correspond to a single sequencing run per sample. To assess reproducibility, biological replicates were independently prepared for each condition, encompassing the full workflow from RNA expression to sequencing (Fig. 4). Genome-wide coverage plots for these replicates are presented in Supplementary Fig. 7. Gene-level expression reproducibility is illustrated in Supplementary Fig. 8."* (lines 740-744).

2.3 Minor remarks

***Title:* Consider rephrasing to highlight the research scope and biological impact.**

The manuscript title has been updated to better reflect the study's scope and findings. The former title, "Cell-free genome-wide transcriptomics through machine learning optimization" *is now replaced with "Active-learning-guided optimization of cell-free systems for genome-wide transcriptomic profiling reveals progressive layers of regulation."* (lines 1-2).

(lines 23-24: **Consider changing to: "Lysate-based cell-free systems (CFS) provide a powerful platform for studying fundamental biological processes and molecular mechanisms".**
As suggested by the reviewer, we replaced *"Lysate-based cell-free systems (CFS) are valuable platform for studying the living and elucidating molecular mechanisms1–3" (lines 23-24)* with *"Lysate-based cell-free systems (CFS) are valuable platform for studying fundamental biological processes and molecular mechanisms[1–3]."* (lines 40-41).

(lines 28-30: Consider rephrasing to: "For example, previous experiments showed that production and metabolic burdens can be untangled by complementing cell-free systems and in vivo measurements".
As suggested by the reviewer, we replaced *"For example, previous experiments showed that production and metabolic burdens can be untangled using cell-free compared with in vivo measurements[4]." (lines 23-24)* with *"For example, previous experiment showed that production and metabolic burdens can be untangled using cell-free compared with in vivo measurements[5]."* (lines 46-47).

Line 31: Remove "and parts, biosensing".
As suggested by the reviewer, we replaced *"CFS provide an open platform ideally suited for protein production, high-throughput prototyping of genetic circuits and parts, biosensing biomanufacturing and gene expression analysis and modelling[5–7]" (lines 30-32)* with *"CFS provide an open platform ideally suited for protein production, high-throughput prototyping of genetic circuits, biomanufacturing and gene expression analysis and modelling[6–8]."* (lines 47-48).

Line 33: **Change "Genome expression" to "Genome-wide expression".**
As suggested by the reviewer, we replaced "Genome expression in living cells is routinely analyzed using RNA-seq[8]" (lines 30-32) with *"RNA sequencing (RNA-seq) is a key method for studying gene expression in living cells[9]."* (lines 52-53).

Supplementary Note 2, Line 54: **Consider adding a sixth category: high NTP-s with low Mg-glutamate levels.**

The supplementary note 2 was incorporated in the results section and the sixth category was added *"Several regions remain underexplored, including combinations of low DNA with elevated NTPs or Mg-glutamate, elevated amino acids with high NTPs, high K-glutamate with either elevated NTPs or Mg-glutamate and elevated NTPs with low Mg-glutamate."* (lines 214-216).

Supplementary Note 3, Line 83: **Change "significative" to "significant".**

The supplementary note 3 was incorporated in the results section and we changed significative" to "significant": *"These changes resulted in a significant increase in mRNA yield in plate 7 (Fig. 2a-b)"* (lines 161-162).

Lines 64-65: **The statement that PEG-8000, amino acids, and spermidine have no impact on mRNA abundance should be rephrased. Given the transcription-centric objective and the tested ranges, modest or interaction-mediated effects could be missed. Please rephrase to: "We did not observe a detectable effect of spermidine, amino acids, or PEG-8000 on mRNA abundance in CSF within the tested ranges". It would also be helpful if the authors could briefly discuss possible reasons for these findings (especially in the context of discordant results for spermidine in the published literature).**
As suggested by the reviewer, we replaced *"Spermidine, amino acid and PEG-8000 seem to have no impact on mRNA abundance in cell-free (Fig. 1e, Supplementary fig. 4)."* (lines 64-65) with *"In contrast, three compounds in the buffer, amino acids, spermidine, and PEG-8000, remained unconstrained by*

the active learning model suggesting that they have little influence on mRNA yield within the tested ranges (Supplementary Fig. 3)." (lines 195-198).

We also discussed the possible reasons of our findings: *"For amino acids this result was expected as they are not required for transcription. Although spermidine has been reported as a critical component for in vitro transcription[30], our data showed no benefit which may be explained by the presence of endogenous polyamines in the lysate that compensate for its absence. Additionally, since PEG-8000 is designed to enhance crowding in the cell-free reaction, the increased concentrations of other components may have mitigated its effects."* (lines 198-203).

***Supplementary Figure 3, Line 101, Supplementary Figure 4, Line 108, Supplementary Note 4, Line 110:* Contain leftover text: "Error! Reference source not found".**
We corrected these errors (lines 32 and lines 39 of supplementary material).

***Lines 65-66:* Replace "the compositions which favored mRNA yields…" with: "most compositions which favored mRNA yields…".**
As suggested by the reviewer, we replaced *"Interestingly, the compositions which favored mRNA yields resulted in low protein yields (Fig. 1f, Supplementary fig. 3)."* (lines 65-66) with *"Most compositions which favored mRNA yields resulted in reduced protein yields, consistent with previous observations[31] (Fig. 3a, Supplementary Fig. 3)."* (lines 207-209).

***Line 70:* Change to "higher levels of Mg-glutamate and NTPs, but lower levels of K-glutamate, amino acids, and PEG-8000…".**
As suggested by the reviewer, we replaced "*The optimized buffer used for our cell-free RNA-seq analysis shows higher Mg-glutamate and NTPs, but lower K-glutamate, amino acids and PEG-8000 compared to the reference buffer (Fig. 1g)."* (l. 69-71) with *"The mRNA-optimized buffer contains higher levels of Mg-glutamate and NTPs, but lower levels of K-glutamate, amino acids, and PEG-8000 compared with the reference buffer (Fig. 3b)."* (lines 225-227).

***Figure 1g:* Include the trade-off buffer composition.**
Fig. 1g in the previous version of the manuscript corresponds to Fig. 3b in the revised manuscript; we included the trade-off buffer composition (line 231).

***Line 94:* Please clarify the term "T7 optimized buffer" (mRNA-optimized buffer?).**
As suggested by the reviewer, we replaced "*T7 Optimized buffer*" with: *"The optimized cell-free reactions were then supplemented with the T7 genome"* (lines 245-246).

***Supplementary Note 5:* The definition of promoter strength based on coverage windows (+12–42 vs. 0–10) seems somewhat arbitrary, it would be useful to justify this choice. Additionally, the discussion on the genomic context remains descriptive, more explicit analysis of sequence motifs or neighboring structural features could add to the mechanistic insight.**
As suggested by the reviewer, we detailed our choice of the coverage window in the legend of Supplementary Fig. 9: *"Bar plot showing normalized promoter strengths derived from genome-wide RNA-seq coverage in the minimalist system. Promoter strength was quantified as the difference between the mean coverage from position +12 to +42 downstream of the transcription start site (TSS) of the annotated promoter and the mean coverage within the first 10 bases (position 0 to +10 relative to the TSS). This windowing strategy was chosen to (i) capture productive elongation downstream of promoter escape while avoiding the immediate promoter-proximal region, which is influenced by transcription initiation dynamics and polymerase pausing, and (ii) provide a local baseline for each promoter, thereby reducing the impact of upstream transcriptional read-through. Negative values were set to zero; this occurred only for the E. coli promoters A1, A3, C and E. Strength values were normalized*

to the maximum observed signal. Bars are colored according to promoter sequence, highlighting the relationship between sequence variation and transcriptional output within the native genomic context. Promoter strengths closely matched previous rankings based on [α-32P]ATP-labeled run-off transcripts1 (Supplementary Table 1), and expanded the analysis to the 18 additional promoters not previously characterized." (lines 62 and lines 74 of supplementary material).

***Supplementary Note 5, Line 138:* Change "Comparison" to Comparison of".**

The content of supplementary Note 5 has been incorporated in the results and the title of Supplementary figure 7 has been corrected (line 49 of supplementary material).

***Lines 113-114:* The authors attribute the 3' coverage skew in vivo to the presence of RNase E, which is absent from CFS. While this is plausible, other E. coli ribonucleases also contribute to global mRNA turnover and could play a role in shaping transcript coverage. The authors might wish to acknowledge this.**

We introduced the fact that RNaseE is one among all possible RNAses that could explain the 3' coverage skew *"One hypothesis for the difference in degradation is the absence of membrane-associated proteins. Among the ribonucleases of E. coli, RNase E is membrane-associated[36,37] and its absence in CFS[1] could account for the observed differences."* (lines 296-298).

***Line 277:* Please clarify the variable "$FluoJove$+" ($FluoRef$+?).**

We corrected this notation by replacing it with "$\boldsymbol{scaling\ factor} = \frac{\boldsymbol{Fluo_{max}}(corr\_buffer, corr\_plate) - \boldsymbol{Fluo_{max}}(\boldsymbol{Ref}-, corr\_plate)}{\boldsymbol{Fluo_{max}}(\boldsymbol{Ref}+, corr\_plate) - \boldsymbol{Fluo_{max}}(\boldsymbol{Ref}-, corr\_plate)}$" (line 494).

***Line 303:* Please explicitly provide the employed GP hyperparameters.**

We now provide this information in the methods: *"Gaussian Processes were tuned for different kernel combinations (White, RBF, and Matérn) and for assumed noise variance (0.01, 0.05, 0.1)"* (lines 566-568).

***Lines 351-352:* It is unclear whether the results obtained from E. coli RNAP were analysed as these results do not appear in the main text.**

The cell-free experiment using purified *E. coli* RNAP was performed (raw data available at NCBI SRA (ID: PRJNA1217614) but not analysed in the final version of the paper due to the poor quality of the RNA-seq results. We removed it from the methods**:** *"Genome-based expression and RNA purification in the minimalist system The T7 genome was expressed using purified T7 RNA polymerase (TranscriptAid T7 High Yield Transcription Kit, Thermo Fisher #K0441) in a 20 µL reaction. After a 2-hour incubation at 37°C, the RNA was treated with DNase I (2 µL) for 15 minutes at 37°C, followed by 2 µL 0.5 M EDTA (pH 8.0) and incubation at 65°C for 10 minutes to stop the reaction. RNA was then purified using the Monarch RNA Cleanup Kit (NEB T2050), polyadenylated (see section 5.18), and sequenced (see section 5.19)."* (lines 624-629).

***Supplementary Figure 10:* I suggest presenting this information in a table rather in a barplot. The number overlay makes the plot cluttered.**

Supplementary Fig. 10 in the previous version of the manuscript corresponds to Supplementary Fig. 14 and contains a table in panel b.

***Supplementary Figure 10, Line 181:* Change "mapping" to "mapping to".**

As suggested by the reviewer, we modified the legend of Supplementary Fig. 14: *"Reads mapping to the E. coli genome predominantly originate from ribosomal RNA present in the lysate."* (lines 121-122 of supplementary material).

Supplementary Figure 10, Line 186: Change "originates" to "originate".

As suggested by the reviewer, we modified the legend of Supplementary Fig. 14: *"Reads mapping to the E. coli genome predominantly originate from ribosomal RNA present in the lysate."* (lines 121-122 of supplementary material).

Line 440: **Change "samtools" to "samtools depth".**
As suggested by the reviewer, We modified the text: *"Base-level coverage was extracted from BAM files using SAMtools depth[50]"* (line 722).

3 Reviewer #3 (Remarks to the Author)

I co-reviewed this manuscript with one of the reviewers who provided the listed reports. This is part of the Nature Communications initiative to facilitate training in peer review and to provide appropriate recognition for Early Career Researchers who co-review manuscripts.

REVIEWERS' COMMENTS

Reviewer #1 (Remarks to the Author):

The authors have addressed my concerns. It's an exciting manuscript.

Reviewer #2 (Remarks to the Author):

The authors have substantially strengthened the manuscript with additional experiments, improved statistical analyses, clearer methodological descriptions, and better overall readability. I have four minor comments remaining:
1) The description of promoter-related findings would benefit from further clarification. Specifically, the text in lines 285-290 is difficult to follow and should be rewritten for improved clarity and flow. In its current form, the connection between coverage profiles and promoter strength is not sufficiently explicit and several statements appear abrupt. It would also strengthen this section to incorporate a brief summary of the results presented in Supplementary Figure 9 and Supplementary Table 1. Finally, please correct the typo on line 287 ("pics" to "peaks").
2) The validation experiment using endogenous E. coli RNA polymerase is introduced for the first time in the Discussion (lines 361-368). Since this constitutes an important experimental result supporting the generalizability of the optimized buffers, it would be more appropriate to present these data in the Results section, with the corresponding Supplementary Material reference.
3) While the authors note that the improvement achieved with the optimized buffer relative to the reference buffer is smaller for E. coli RNAP than for T7 RNAP (~4-fold vs ~20-fold), this difference is substantial and could be discussed more explicitly. A similar point also applies to the trade-off buffer. These observations suggest that both buffer conditions may be at least partially specific to the T7 transcription system, and that the current evidence supporting broader generalizability remains limited.
4) References to the Supplementary Material are still broken in the main text, appearing as "Error! Reference source not found" instead of proper pointers. Please ensure this issue is resolved in the final version, as it significantly affects readability.

Reviewer #3 (Remarks to the Author):

I co-reviewed this manuscript with one of the reviewers who provided the listed reports. This is part of the Nature Communications initiative to facilitate training in peer review and to provide appropriate recognition for Early Career Researchers who co-review manuscripts.

We have addressed all remaining comments from Reviewer 2:
1) We expanded the description to improve clarity and readability about the "Promoter-related findings" (lines 235–245)
2) We retained in the discussion section the data about *E. coli* RNA polymerase as it opens avenues for future research, while providing a more thorough description (lines 299–307)
3) We tempered the claims regarding generalizability to novel RNA polymerases (line 304)
4) We corrected the broken links.
